# Practical Considerations and Limitations of Using Leaf and Canopy Temperature Measurements as a Stomatal Conductance Proxy: Sensitivity across Environmental Conditions, Scale, and Sample Size

**DOI:** 10.34133/plantphenomics.0169

**Published:** 2024-04-15

**Authors:** Ismael K. Mayanja, Christine H. Diepenbrock, Vincent Vadez, Tong Lei, Brian N. Bailey

**Affiliations:** ^1^ Department of Biological Systems Engineering, University of California, Davis, Davis, CA, USA.; ^2^ Department of Plant Sciences, University of California, Davis, Davis, CA, USA.; ^3^ French National Research Institute for Sustainable Development (IRD), UMR DIADE, University of Montpellier, Montpellier, France.

## Abstract

Stomatal conductance (*g_s_*) is a crucial component of plant physiology, as it links plant productivity and water loss through transpiration. Estimating *g_s_* indirectly through leaf temperature (*T_l_*) measurement is common for reducing the high labor cost associated with direct *g_s_* measurement. However, the relationship between observed *T_l_* and *g_s_* can be notably affected by local environmental conditions, canopy structure, measurement scale, sample size, and *g_s_* itself. To better understand and quantify the variation in the relationship between *T_l_* measurements to *g_s_*, this study analyzed the sensitivity of *T_l_* to *g_s_* using a high-resolution three-dimensional model that resolves interactions between microclimate and canopy structure. The model was used to simulate the sensitivity of *T_l_* to *g_s_* across different environmental conditions, aggregation scales (point measurement, infrared thermometer, and thermographic image), and sample sizes. Results showed that leaf-level sensitivity of *T_l_* to *g_s_* was highest under conditions of high net radiation flux, high vapor pressure deficit, and low boundary layer conductance. The study findings also highlighted the trade-off between measurement scale and sample size to maximize sensitivity. Smaller scale measurements (e.g., thermocouple) provided maximal sensitivity because they allow for exclusion of shaded leaves and the ground, which have low sensitivity. However, large sample sizes (up to 50 to 75) may be needed to differentiate genotypes. Larger-scale measurements (e.g., thermal camera) reduced sample size requirements but include low-sensitivity elements in the measurement. This work provides a means of estimating leaf-level sensitivity and offers quantitative guidance for balancing scale and sample size issues.

## Introduction

In order for carbon dioxide (CO_2_) to diffuse into internal leaf cells where photosynthesis takes place, plants expose these cells to the ambient environment by opening stomatal pores. In doing so, the moist internal cells without an epidermal layer are exposed to the dry air, which results in high rates of water loss due to evaporation. Thus, the rates of water loss and rates of carbon gain in plants are generally observed to be tightly linked [1]. Stomata tend to close in conditions of high water demand from the atmosphere such as when ambient air temperature (*T*_air_) is high, relative humidity (*Rh*) is low, or the soil is dry due to low water supply [2]. At some critical point, this stomatal closure can restrict the flow of CO_2_ into the plant such that rates of photosynthesis, and ultimately growth and yield, are negatively affected, which is commonly termed “water stress.” Some plants possess an adaptation known as the “limited transpiration trait,” which allows them to limit water loss through transpiration at a high vapor pressure deficit (VPD). This trait contributes to early-season water conservation and can ultimately lead to improved yield under drought conditions [3–5]. While there are other nonstomatal factors, such as Rubisco activity and carboxylation efficiency, that can negatively affect plant productivity in the presence of drought [6], stomatal conductance (*g_s_*), which quantifies the rate of gaseous exchange between the interior of the leaf and the surrounding air through stomata, is often used as a convenient indicator of the degree to which plant productivity is limited by insufficient water supply or excessive evaporative demand.

Measurement of plant water status has become increasingly important in agricultural applications including crop breeding and irrigation management. Crop breeding programs are progressively considering traits related to plant water status in the context of drought tolerance, which has been an increasingly prevalent breeding target due to concerns surrounding the increased frequency and severity of drought periods attributed to climate change and the need to ensure food, fodder, and nutritional security [7,8]. Crop growth models may also benefit from measurements of crop water status for developing and parameterizing models to predict phenology and yield in response to drought [9]. In irrigation management, measurement of water status is a critical component of precision irrigation, which aims to apply the appropriate amount of water when required by the crop, with the goal of reducing water consumption while sustaining productivity [10,11].

In small field trials, stomatal conductance can be feasibly quantified through direct measurement using a porometer or infrared (IR) gas analyzer [12], which is relatively accurate but becomes prohibitively laborious at large scales. Therefore, leaf or canopy temperature has been widely used as a proxy measure of plant water status, which leverages the fact that leaf temperature (*T_l_*) is inversely related to *g_s_*. The closure of stomata (reduction in *g_s_*) decreases the rate of transpiration or water loss from the leaf through evaporation, which decreases the latent cooling effect and leads to an increase in *T_l_* [13]. However, ambient environmental variables (e.g., air temperature, humidity, and incident radiation) can also strongly affect *T_l_* irrespective of a change in *g_s_* [14], and thus, the impact of variable environmental conditions must be separated to isolate the effect of plant water status on *T_l_* measurement. Consequently, attempts have been made to normalize *T_l_* measurements, such as development of the crop water stress index (CWSI), to generate a standardized relationship between *T_l_* and plant water stress at a particular VPD [15–18]. Nonetheless, Poirier-Pocovi and Bailey [19] showed that the CWSIs are often not effective at removing the effect of ambient weather conditions, whereby the CWSIs are more sensitive to variables such as wind speed (*U*) than *g_s_*. In addition, an explicit understanding of the quantitative effect of ambient conditions on the *T_l_* − *g_s_* relationship and the behavioral response of *T_l_* to changes in *g_s_* would be beneficial for breeders, physiologists, and irrigation managers to gauge the appropriate time of the day and sample size for conducting *T_l_* measurements, and ultimately to understand limitations inherent in temperature-based estimation of *g_s_*.

The objective of this study was to provide quantitative guidance on how changes in *T_l_* correspond to observed changes in *g_s_* and to better understand the limitations in these measurements in terms of variation in the sensitivity of *T_l_* to *g_s_*, confounding effects of environmental conditions, scale of measurement aggregation, and sample size. This involved (a) developing condensed graphs and mathematical equations for quantifying the responsiveness of *T_l_* to *g_s_*, (b) determining both the ideal and undesirable ambient conditions for *T_l_* measurements when predicting plant water status, (c) and developing a case study to illustrate the application of *T_l_* − *g_s_* sensitivity in temperature-based water status quantification in the field. The case study was also used to analyze and recommend a suitable sample size when collecting *T_l_* measurements.

## Materials and Methods

### Theoretical framework

#### Quantifying sensitivity of leaf temperature to stomatal conductance

The sensitivity of leaf temperature *T_l_* (dependent variable) to stomatal conductance *g_s_* (independent variable) was explored as ambient environmental conditions vary, which included air temperature, air relative humidity, wind speed, and solar radiation flux. Mathematically, *S* was defined as the derivative of *T_l_* with respect to *g_s_* at constant ambient conditions:

$$S=\frac{{\mathrm{dT}}_{l}}{{\mathrm{dg}}_{s}}$$ (1)

**Fig. 1.**
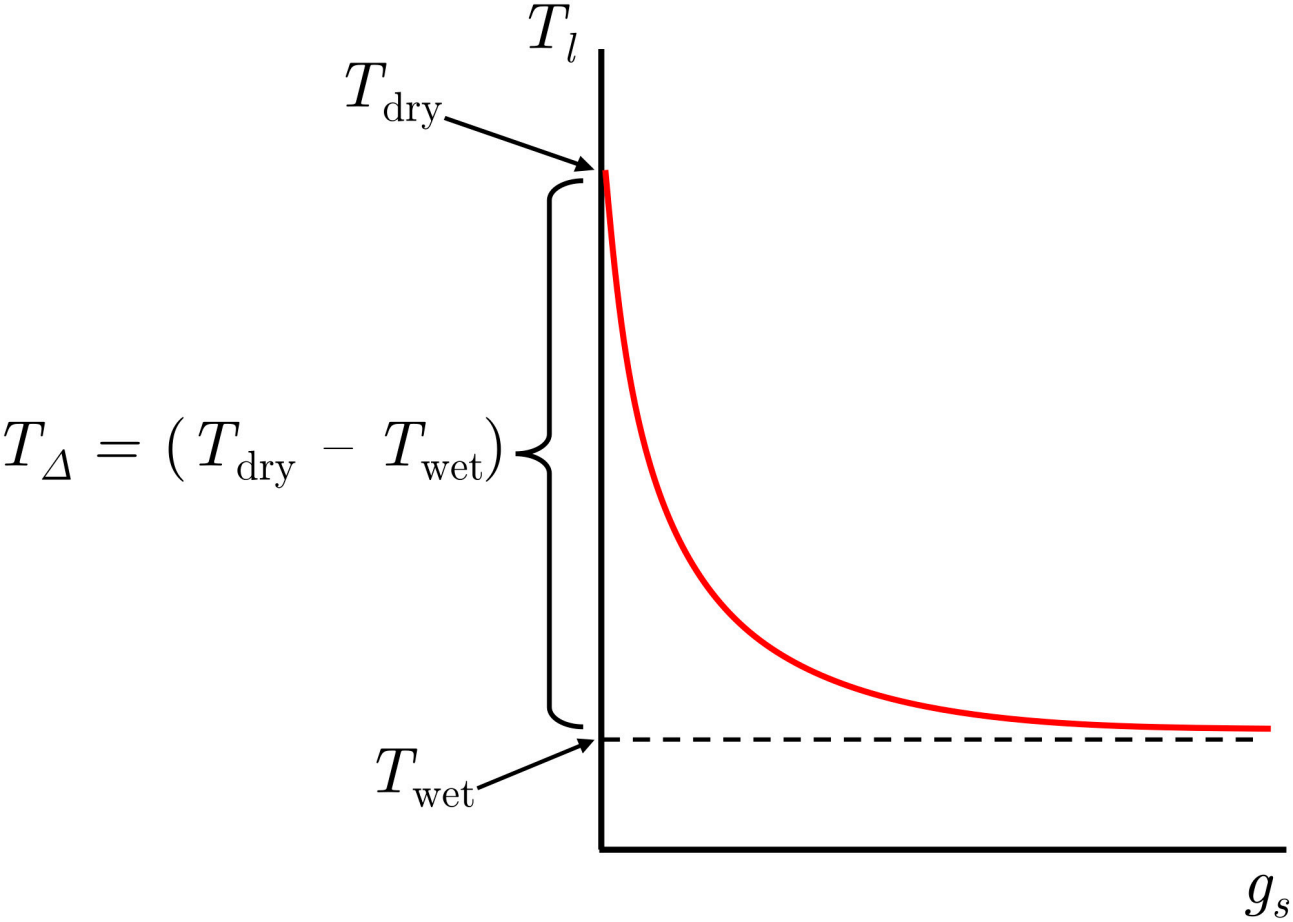
Typical response of leaf temperature (*T_l_*) to varying stomatal conductance (*g_s_*). As *g_s_* increases, the sensitivity of *T_l_* to *g_s_* (or slope of the curve) decreases. *T*_dry_ is the leaf temperature when *g_s_* is 0, *T_wet_* is the leaf temperature of an equivalent wet surface with unlimited supply of free water, and *T*_Δ_ is the difference between *T*_dry_ and *T*_wet_.

A typical response of *T_l_* to variation in *g_s_* is illustrated in Fig. 1 (see also [20]). Considering constant ambient conditions, the maximum *T_l_* (denoted as *T*_dry_) is theoretically attained when stomata are fully closed (*g_s_* → 0) and transpiration has ceased (assuming minimal water loss from epidermal cells). The opening of stomata (increase in *g_s_*) gradually decreases *T_l_* via an increase in latent cooling, until a point is reached where a negligible decrease in *T_l_* (denoted as *T*_wet_) can be observed regardless of a further increase in *g_s_*. Thus, sensitivity decreases continuously as *g_s_* increases and asymptotes to a value of 0.

While the sensitivity, *S*, provides a useful means of quantifying the responsiveness of *T_l_* to *g_s_*, *S* varies considerably as *g_s_* changes. To derive bulk parameters that describe the entirety of the *T_l_* − *g_s_* curve for given ambient conditions, a simplified equation based on a first-order system response to a step function input [21] was used to describe this curve

$$T_{l}=T_{\text{wet}}+T_{\Delta }\;\text{exp}\left(-\frac{g_{s}}{c}\right)$$ (2)

where *T*_Δ_ = *T*_dry_ − *T*_wet_, and *c*, which we call the “stomatal constant,” defines the rate at which *T_l_* approaches *T*_wet_ with increasing *g_s_*. *c* is analogous to the time constant of a first-order dynamical system and has the same units as *g_s_*. Physically, *c* is the stomatal conductance at which *T_l_* is 63% between *T*_dry_ and *T*_wet_ (i.e., (*T*_dry_ − *T_l_*)/*T*_Δ_ = 0.63). The change in *T_l_* with an incremental change in *g_s_* is largest when *T_l_* ≈ *T*_dry_, and decreases as *g_s_* increases.

The derivative of Eq. 2 with respect to *g_s_* yields an analytical approximation of *S*, provided *T*_dry_, *T*_wet_, and *c* are known for particular ambient environmental conditions

$$S=\left|\frac{-T_{\Delta }}{c}\;\text{exp}\left(-\frac{g_{s}}{c}\right)\right|$$ (3)

which takes *g_s_* as the input variable. Alternatively, *S* can be calculated using *T_l_* as the input variable by making $T_{\Delta }\;\text{exp}\left(-\frac{g_{s}}{c}\right)$ the subject of Eq. 2 and substituting into Eq. 3

$$S=\left|\frac{T_{\text{wet}}-T_{l}}{c},\;\right|$$ (4)

Environmental conditions that maximize *T*_Δ_ will amplify the overall change in *T_l_* as *g_s_* is varied from a low to a high value. Conditions that decrease the value of *c* increase the rate at which *T_l_* approaches *T*_wet_ as *g_s_* increases, which increases *S* at low *g_s_* and decreases *S* at high *g_s_*.

### Modeling of leaf surface temperature across scales

Determination of *S* by direct measurement is extremely challenging, as it requires simultaneous measurement of *T_l_* and *g_s_* for systematically varied *g_s_* across a wide range of environmental conditions. Alternatively, mechanistic equations based on first principles are available for calculation of *T_l_* as a function of *g_s_* and other environmental variables, which have been demonstrated to closely replicate measured values [17,22].

#### Leaf-level energy balance equation

A standard surface energy balance model was used to calculate the surface temperatures of leaves and surrounding objects [23], and ultimately *S*. Briefly, this involves determining the budget of surface energy fluxes, which was assumed to consist of a balance of net absorbed all-wave radiation, emitted longwave radiation, sensible heat exchange, and latent heat exchange. This equation can be iteratively solved for the surface temperature that balances the energy budget. A detailed description of the full energy balance model solution procedure can be found in Supplementary S1.

#### Three-dimensional simulation of surface temperature

The use of a simulated environment based on biophysical models enables the systematic variation of environmental conditions to replicate different levels of temperature measurement aggregation and their impact on the *T_l_* − *g_s_* relationship, which would not be possible experimentally in the field. To allow for the simulation of the various scales of temperature measurement considered in this work, a model that can accurately predict the three-dimensional (3D) distribution of surface temperature from subleaf through canopy scales is needed. Several 3D biophysical models have been developed, which solve the leaf-level energy balance to predict the distribution of surface temperature, such as ARCHIMED [24], GroIMP [25], HydroShoot [26], and others. However, these models do not simulate measurements from remote sensors such as an IR thermometer or thermal camera. 3D models for remote sensing applications have been developed to simulate remote temperature measurements such as DART-Lux [27] and LESS [28], but they do not represent the biophysics needed to predict accurate subleaf-scale distributions of temperature.

In this study, the Helios 3D modeling framework (version 1.2.56) [29] was chosen for the simulation of temperature measurements in response to environmental variation. Helios was chosen due to its unique ability to simulate temperature measurements at various scales based on 3D biophysical modeling of subprocesses contributing to these measurements. Within the Helios framework, triangular and rectangular meshes represent the 3D geometry of leaves, stems, fruit/grain, and the ground [29]. The surface energy balance described above was applied to each of these geometric elements in the mesh to determine the 3D distribution of temperature. Radiative fluxes *R_SW_* and *R_LW_* needed as inputs to the energy balance equation were determined using the reverse ray tracing model of [30], which simulates radiative transport through the model domain. Other inputs were specified as described in Supplementary 1. Aerial thermal images were simulated by combining the Helios radiation and energy balance models with a thin-lens camera model adapted from [31] (further described in Supplementary 2).

#### Scales of simulated measurement aggregation

**Fig. 2.**
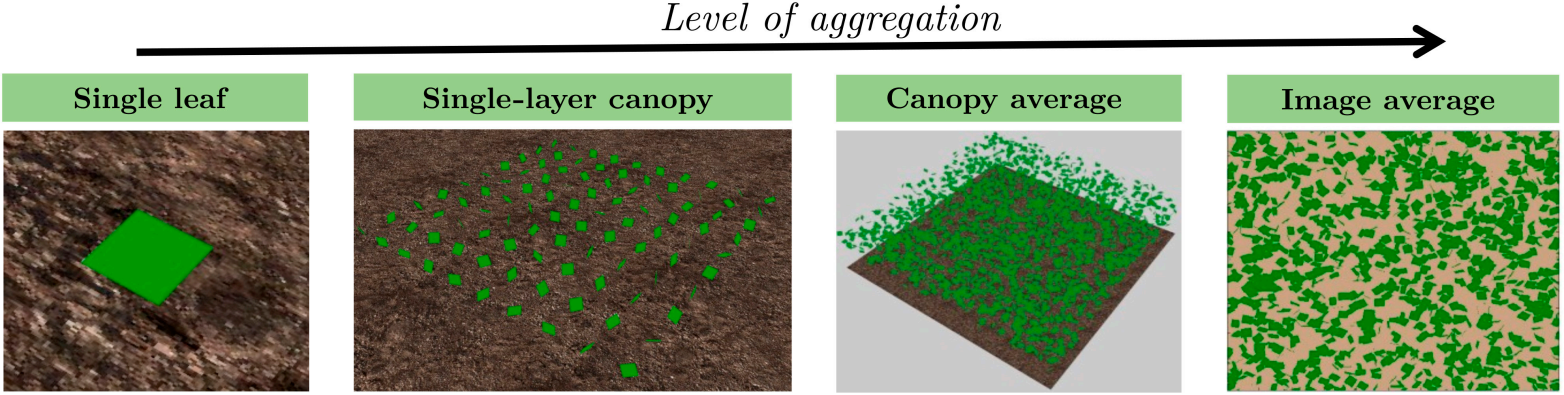
Schematic diagram showing the different levels of aggregation used in simulated *T_l_* measurement, scaling with an increase in the level of complexity from a single isolated leaf parallel to the ground, to a single-layer canopy of leaves randomly orientated with no self-shading, to a canopy average, and an image average representative of an aerial thermal image of the homogeneous canopy.

Various levels of aggregation in simulating *T_l_* measurements were considered in this study, which involved scaling from a single leaf to a canopy level as illustrated in Fig. 2. Sub-grouping the canopy structures into levels with increasing complexity allowed for the explicit understanding of sensitivity responses for each type of measurement. The levels of aggregation were (a) an isolated, 0.1 × 0.1  m^2^ horizontal leaf raised 0.3  m from the ground referred to as “single leaf” (since the single leaf is parallel to the ground, it was assumed to have a uniform *T_l_* corresponding to spatially uniform *g_s_* and ambient conditions); (b) a single-layer canopy with no self-shading, which is composed of widely spaced leaves (total leaves = 900) along a plane perpendicular to the ground, and randomly oriented following a spherical angle distribution (similar to the single leaf, each leaf in the single-layer canopy was assumed to have uniform *T_l_* since there is no shading, but different *T_l_* across leaves because they have different orientations to the sun and the ground; therefore, a single simulated *T_l_* measurement was taken for each leaf); (c) a homogeneous canopy (referred to as “canopy average”) that repeats indefinitely in the horizontal direction, follows a spherical leaf angle distribution, and has a leaf area index (LAI) of 1 and a height of 1 m (due to self-shading and leaf orientation within the canopy, *T_l_* is nonuniform both within each leaf and across different leaves; to account for this nonuniformity, each leaf was divided into 15 by 15 subdivisions across the leaf’s length and width, which represents 225 *T_l_* data points per leaf generated for the corresponding *g_s_* and ambient conditions); and (d) image average, which represents an aerial thermal image of the homogeneous canopy, serving as a convenient and efficient method for measuring *T_l_* due to the impracticality of measuring the entire canopy. Simulations of in-field measurements were performed using either an IR thermometer or a thermal camera. The simulated thermal camera used in this study had a 512 × 512 resolution, 20^∘^ horizontal field of view (HFOV), and 5-m distance from the ground to the camera focal plane, allowing it to capture a field size of 1.5 × 1.5  m^2^ in the thermal images. Each pixel of the thermal camera (total pixels  = 262,144) represents a *T_l_* data point. These individual *T_l_* pixel readings were averaged to calculate the mean *T_l_* and the associated *g_s_* for the specific leaves captured by the thermal camera. The methodology for developing the thermal image was the same for both the IR thermometer and the thermal camera, with the distinction that the IR thermometer operates at a smaller scale compared to the thermal camera. The process of acquiring IR thermometer readings is comprehensively outlined in the case study section where its practical implementation is also demonstrated. For all these cases, the sun was considered to be at its zenith unless otherwise specified.

LAI impacts the proportion of sunlit or shaded leaves and the fraction of ground visible to the thermal camera. An analysis was conducted to determine how the *S* in canopy average and image average is affected by an increase in LAI from 0.5 to 1.5, and then to 3. The increase in LAI within the Helios framework is accomplished by adjusting the number of leaves in a unit ground area while still adhering to a spherical leaf angle distribution until the desired LAI is reached (0.5, 1.5, and *3*). This adjustment is automatically executed in Helios through the “Canopy Generator” plug-in.

### Case study: Detecting differences in stomatal traits among sorghum genotypes in a breeding trial

#### Background

A case study that focused on the example problem of using temperature measurements to phenotype stomatal traits in a breeding trial was developed. Measurement of stomatal-related traits is generally limited by the throughput of the measurement, as breeding trials commonly consist of hundreds to thousands of small plots with hundreds of different genotypes in total that require characterization. Temperature-based measurement of stomatal traits can be much faster than direct measurement. The measurement scale of leaf temperature (*T_l_*) varies based on the instrument. On one hand, point-scale instruments such as thermocouples provide a focused temperature reading at a specific point on a leaf surface, which gives an accurate, direct measurement of temperature at that point. However, the reliability of point measurements is highly dependent on the sample size collected, as sufficient sample size is needed for measurements to be representative of the whole plant or canopy. In practice, however, the sample size is typically limited to a few *T_l_* measurements, which raises questions regarding the representativeness of the measurements for the whole plant or canopy. On the other hand, instruments that provide spatially averaged measurements of temperature, such as IR thermometers or thermal cameras, sample a section of a canopy, which allows measurement on a larger scale. However, the spatial average is influenced by the sensor viewing angle, which determines the fraction of shaded leaves included in the spatially averaged *T_l_* measurement. The LAI of the canopy also determines the amount of ground surface area included within the measurement. Each of these issues may have an effect on the sensitivity *S* of the measurement and ultimately will determine the degree to which a measurement of *T_l_* will be able to detect differences in plant water status via *g_s_*.

Virtual 3D sorghum canopies were developed in Helios using manual geometric measurements obtained in the field for four genotypes (described below). The model was used to evaluate whether statistically significant differences in the stomatal conductance of each genotype could be detected based on measurements of *T_l_* at different levels of aggregation in *T_l_* measurement, sun direction, and ambient conditions. The effect of sample size on the ability to distinguish between genotypes was also evaluated.

#### Sorghum field trial measurements and model setup

Four genetically and geographically diverse sorghum lines, which are among the parents of the sorghum Nested Association Mapping panel ([32], Table S1), were grown at the University of California, Davis Agronomy fields (Davis, CA, USA). Each genotype was planted in two-row plots of size 1.22 × 3.05  m^2^ each. Two months after planting, the average stand count was 25 plants per plot (50 per genotype) when averaged across all genotypes, and the realized average spacing was 0.12  m ×  0.76  m (between plant × between row). Also, geometric measurements of the sorghum canopy, including leaf length, leaf width, stem height, panicle height, panicle diameter, and the number of leaves, were obtained using a tape measure. These measurements were based on an average of 10 samples per genotype conducted on different plants within the same plot. Leaf length was defined as the distance from the base to the apex of the leaf, leaf width as the widest part of the leaf perpendicular to the midrib, stem height as the vertical distance from the ground to the stem-panicle contact, panicle height as the vertical extent of the panicle, and panicle diameter as the horizontal measurement across the widest part of the panicle.

The leaf and soil spectral properties were measured using a combination of a field spectroradiometer (PSR+3500) and a reflectance/transmittance integrating sphere (Spectral Evolution Inc., Haverhill, MA, USA). The measured spectral reflectance and transmittance distributions were integrated across two solar bands—photosynthetically active radiation (PAR; ≤700 nm) and near-infrared radiation (NIR; >700 nm)—to calculate total radiative properties and incorporated in the sorghum canopy, which included leaf PAR reflectivity (0.1157), leaf NIR reflectivity (0.417), leaf PAR transmissivity (0.039), leaf NIR transmissivity (0.4441), ground PAR reflectivity (0.1069), and ground NIR reflectivity (0.2745). Furthermore, 30 samples of *g_s_* and corresponding *T_l_* were collected from healthy, fully expanded sunlit leaves for each genotype in the field using a porometer (LI-600, LI-COR Biosciences, Lincoln, NE, USA).

**Fig. 3.**
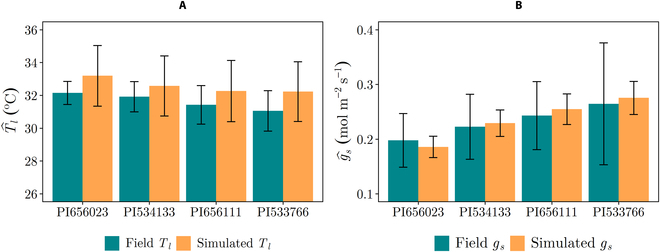
Field and simulated data for (A) *T_l_* and (B) *g_s_* averaged across each genotype. The simulated *T_l_* and *g_s_* are in an acceptable range with measured field data, which shows that the developed sorghum geometry can reliably produce acceptable simulations. The hat operator denotes a spatial average.

A sorghum canopy geometry was then generated in Helios for each genotype by adjusting parameters in the “Canopy Generator” plug-in based on the recorded geometric measurements, stand count, and realized plant spacing. Each genotype was replicated in the simulations five times, whereby each sorghum plant had randomly assigned stem bending angle, stem direction, and leaf azimuth orientations to account for the structural variations between different genotypes and replicates as well. Each leaf was divided into 50 × 10 subunits that were used as the basis of modeled fluxes to fully resolve leaf shadows [33]. The stomatal conductance measurements were incorporated within the model by adjusting the *E_m_* parameter in the *g_s_* model (see Supplementary 1) such that simulated *T_l_* and *g_s_* for each genotype reasonably matched field porometer measurements as shown in Fig. 3. *E_m_* is the maximum transpiration rate as *VPD* approaches infinity and thus effectively sets the maximum stomatal conductance value (higher *E_m_* leads to higher *g_s_* at constant light and VPD). The root mean square error between the simulated and the field measurements was 0.95^∘^C and 0.011  mol  m^−2^  s^−1^ for *T_l_* and *g_s_*, respectively. Also, a hypothetical genotype “*X*” was added to the study to include a genotype with very high *g_s_* values, which was not observed in the four field genotypes considered (see Table S2).

In addition, the environmental conditions of the simulated genotypes in Helios were also matched with the field conditions measured by the porometer, which were *T*_air_ (^∘^*C*) of 30.8 ±  0.2, 30.9 ±  0.2, 30.9 ±  0.2, and 31.0 ±  0.3 and *Rh* of 0.40 ±  0.02, 0.41 ±  0.02, 0.42 ±  0.02, and 0.42 ±  0.02 for genotypes “PI656023,” “PI534133,” “PI656111,” and, “PI533766,” respectively. Also, wind speed data were retrieved from the Davis station of the California Irrigation Management Information System (CIMIS; https://cimis.water.ca.gov) for measurements taken on 2022 July 21 at 11:00 AM.

#### Simulated T_l_ measurement and ambient conditions

The smallest scale of measurement considered in this study was a point temperature measurement (e.g., a thermocouple). Based on the simulated temperature field, a point/thermocouple measurement was assumed to correspond to calculated *T_l_* for a single leaf mesh triangle (there were roughly 1,000 triangles per leaf). For each genotype, 60,000 simulated thermocouple readings were recorded per replicate, considering only sunlit leaves (*η* > 0.5; see Eq. 6). A thermal camera was the largest scale of measurement considered and consisted of 512 × 512 pixels (each pixel representing a *T_l_* reading) averaged into a single image for each genotype per replicate. The camera had a 20^∘^ HFOV and was positioned at a distance of 5 m from the ground, allowing it to capture a field size of 1.5 × 1.5  m^2^ in the thermal images. In addition, an intermediate scale of measurement was simulated representative of an IR thermometer. Comparing the typical spot size of IR thermometers (0.05  m) to the pixel size of the simulated thermal camera shows that about 289 (17 × 17) pixels of the thermal camera make up one spot size of the IR thermometer. A single thermal image therefore produces 900 IR thermometer *T_l_* measurements.

Actual environmental data from the CIMIS weather station in Davis (described above) was selected for a range of ambient conditions, which was used to assess simulated *T_l_* measurements at each level of aggregation with varying environmental conditions, which included favorable conditions with high *S* (*T*_air_ = 40.3^∘^*C*,  *Rh* = 0.18, *U* = 2.77  m  s^−1^) and unfavorable conditions with a low *S* (*T*_air_ = 14.1^∘^*C*,  *Rh* = 0.75, *U* = 4.74  m  s^−1^). Both the favorable and unfavorable conditions were selected at 1 p.m. when the sun was near its maximum elevation angle on 2022 November 6 and 2022 April 14, respectively. For illustration, Fig. 7 shows a simulated aerial thermal image for the sorghum genotypes used in this case study under favorable and unfavorable conditions. It should be noted that the selection of these dates for favorable and unfavorable conditions was not based on whether they aligned with the sorghum growing season. Instead, the focus was on obtaining extreme ends of the ambient conditions from real weather data to use in the analysis.

To understand the effect of sun direction on *T_l_* measurements, a day with minimal change in environmental conditions across time was chosen from CIMIS to isolate the effect of ambient conditions from the direction of the sun. As such, data recorded on 2022 August 21 were considered, encompassing 3 h, which included (a) 1 p.m., when the sun is 0^∘^ from the thermal camera (*T*_air_ = 31.1^∘^*C*,  *Rh* = 0.29, *U* = 4.78  m  s^−1^); (b) 2 p.m., when the sun is 15^∘^ from the thermal camera (*T*_air_ = 31.3^∘^*C*,  *Rh* = 0.32, *U* = 4.96  m  s^−1^); and (c) 3 p.m., when the sun is 30^∘^ from the thermal camera (*T*_air_ = 31.7^∘^*C*,  *Rh* = 0.31, *U* = 4.56  m  s^−1^).

### Data analysis

#### Simple equations for estimating sensitivity, S

Mathematical models for estimating *c*, *T*_wet_, and *T*_Δ_ (which we call *S*-parameters) as a function of ambient weather, which are the input parameters used in Eqs. 3 and 4, were developed using the graphical method to provide a way to readily approximate *S*. The general form of the mathematical model for deriving the S-parameters is given in Eq. 5, which takes ambient conditions as inputs (derivation described in Supplementary 3).

$$\begin{array}{c} S-\text{parameter}=\\ \left[\left(c_{1}U+c_{2}\right)T_{\text{air}}+c_{3}U+c_{4}\right]\mathrm{Rh}+\left(c_{5}U+c_{6}\right)T_{\text{air}}+c_{7}U+c_{8} \end{array}$$ (5)

where *c*_1_ to *c*_8_ are empirical coefficients generated from linear fitting. *S*-parameter could be either *c*, *T*_wet_, or *T*_Δ_.

The reliability of Eq. 5 in terms of approximating *S*-parameters was quantified by using the coefficient of determination (*R*^2^), which is a measure of the proportion of variation from the predicted dataset that is estimated from the simulated dataset.

#### Statistical tests

All data visualization and analysis was conducted using RStudio 2022.02.0. Pre-analysis showed that simulated *g_s_* and *T_l_* data generally had a skewness value of < − 1.1; therefore, analysis of variance (ANOVA) could not be used since the data was not normally distributed. In this case, nonparametric tests, which do not make assumptions about the distribution of the population from which the sample is drawn were utilized. A Kruskal–Wallis test was conducted across genotypes for each set of ambient conditions to determine whether the differences in *T_l_* and *g_s_* across genotypes were statistically significant. When a significant main effect was found, a post-hoc test using Dunn’s test was used to determine where the difference of the means lies in the sorghum genotypes at a 95% confidence level (*P* ≤ 0.05). Also, bar plots are shown only for those instances where there was a significant difference between genotypes [34].

The sample size used for the simulated thermocouple (60,000 × 5) and IR thermometer (900 × 5) measurements is theoretical and improbable to achieve in the field, making it imperative to investigate the effect of *T_l_* using realistic sample sizes employed in the field (<100). For this analysis, *T_l_* from the thermocouple and IR thermometer for favorable conditions were randomized to eradicate bias, and then averages of 10, 20, 50, and 100 samples were drawn for each genotype. In addition, the sample sizes for the thermocouple and IR measurements required to achieve a statistical power of 0.8 and 0.9 were calculated, which was attained by randomly drawing a sample size from two pairs of genotypes 1,000 times. It was then determined how many times the two genotypes were significantly different at *P* ≤ 0.05 (Mann–Whitney *U* test). The statistical power was computed as the ratio of the number of times the two genotypes were significantly different from the total number of realizations (1,000). The process was repeated for all combinations of genotypes, and the sample sizes required to achieve statistical powers of 0.8/0.9 were interpolated and recorded.

#### Analysis of 3D simulation data

The single-layer and homogeneous canopies in this study had isotropic leaf angle distributions, indicating that approximately half of the total leaf area within the canopy is projected in the direction of the sun at any instant (i.e., *G*-function value of 0.5). As a result, the canopy-averaged absorbed radiation flux was half of the total incoming flux. Peak all-wave solar radiation under clear skies is typically around 1,000  W  m^−2^. This study assumed the incoming radiation to be 1,000 W  m^−2^, which corresponded to an average absorbed flux by leaves of 300 W  m^−2^ which accounts for the *G*-function of 0.5 and an all-wave leaf absorptivity of about 0.6. Also, shaded leaves indirectly receive solar radiation through diffuse radiation scattered by the atmosphere and other leaves, which was assumed to be 50 W  m^−2^.

The leaf sunlit fraction (*η*) was used to differentiate between sunlit and shaded leaves and is defined as

$$\eta =\frac{\text{Actual}\;R_{\mathrm{SW}}}{\text{Unobstructed}\;R_{\mathrm{SW}}}$$ (6)

This index quantifies the ratio of the actual solar radiation flux received by a leaf given its orientation and possible shading by other leaves (*R_SW_*) to the unobstructed solar radiation flux the leaf would have received if it were fully exposed to sunlight (i.e., unshaded). The unobstructed flux is calculated as the absolute value of the dot product between the sun direction vector and the leaf normal vector, multiplied by the above-canopy radiation flux and the leaf absorptivity. Leaves with *η* values greater than 0.5 were classified as sunlit leaves, while leaves with *η* values below 0.5 were classified as shaded.

Scaling of *S* from a single leaf to a canopy level was estimated using a two-leaf model [35,36], whereby *S* for a canopy is the weighted contribution of the sunlit and shaded leaves as shown in the equation:

$$S=f_{\text{sun}}S_{\mathrm{sun}}+f_{\text{shade}}S_{\text{shade}}$$ (7)

where *f*_sun_ is the fraction of sunlit leaves, *f*_shade_ is the fraction of shaded leaves, *S*_sun_ is the *S* of a single sunlit leaf, and *S*_shade_ is the *S* of a single shaded leaf. At a canopy level, other factors such as a change in LAI and movement of the sun (angle between the thermal camera and sun) were also investigated since they have an effect on *S*.

## Results

### Theoretical analysis of sensitivity (*S*)

#### Sensitivity of single leaves in response to changing environmental conditions

**Fig. 4.**
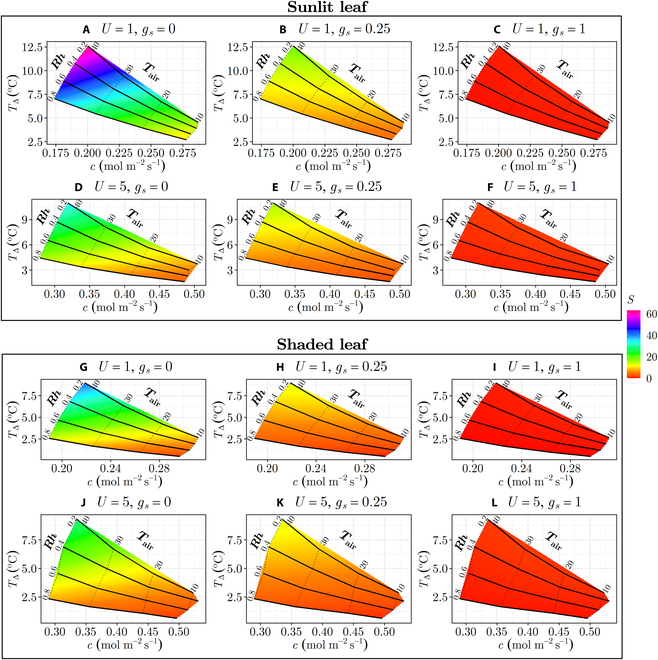
Variation in the sensitivity (*S*) of leaf temperature to stomatal conductance with changes in ambient conditions and stomatal conductance (*g_s_*). Each subplot represents different combinations of relative humidity (*Rh*) ranging from 0.2 to 0.8 and air temperature (*T*_air_) varying from 10 to 40^∘^C. The columns correspond to different *g_s_* ranging from 0 to 1  mol  m^−2^  s^−1^. (A to F) Conditions for a sunlit leaf (*R_SW_* = 300  W  m^−2^). (G to L) Shaded leaf (*R_SW_* = 50  W  m^−2^). The wind speed (*U*) remains constant within each row, with (A) to (C) and (G) to (I) having *U* of 1  m  s^−1^, and (D) to (F) and (J) to (L) having *U* of 5  m  s^−1^. To interpret this plot, a subplot is selected based on ambient radiation level, wind speed, and expected stomatal conductance. The *S* value can be determined by matching the surface color at the given ambient air temperature and humidity to the associated value in the color bar.

The sensitivity plots (*S*-plots) presented in Fig. 4 provide visual representations of how *S* values are affected by variations in ambient conditions and *g_s_* for both a shaded and sunlit single leaf. The plots clearly indicate that larger *S* tended to occur when there was an increase in *T*_Δ_ (i.e., the difference in *T_l_* between 0 and maximal *g_s_*) and a decrease in *c* (i.e., lower overall rate of decline in *T_l_* with increasing *g_s_*). The analysis revealed that *S* is largest under the following conditions: high net radiation flux, high VPD, low boundary layer conductance (such as when wind speed is low), and low stomatal conductance. The response of sensitivity to each of these variables is described independently below.

Net radiation flux (*R_n_*): *S* increases when *R_n_* increases because it provides additional energy that can potentially be dissipated by latent cooling, which is regulated by *g_s_*. This occurs as the surplus energy is utilized to drive the evaporation of water from the leaf surface, leading to a cooling effect [13]. As a result, an increase in *R_n_* enhances the sensitivity. When *R_n_* is low, *T_l_* is generally close to *T*_air_, and sensible and latent heat fluxes are correspondingly low. This means that there is little potential for *g_s_* to affect *T_l_*, hence resulting in a low *S*. As such, *S* will be higher in sunlit leaves since they tend to have higher *R_n_* than shaded leaves.

VPD: Increasing VPD amplifies the latent heat flux term relative to the sensible heat flux term (Eq. S1), which increases the potential of *g_s_* to affect *T_l_*, and thus tends to increase *S*. VPD can be increased by either increasing *T*_air_ or decreasing *Rh*, or by increasing *T_l_* relative to *T*_air_ [17].

Boundary layer conductance (*g_H_*): Interpretation of the effect of boundary layer conductance on *S* is complicated by the fact that it appears both in the sensible and latent heat flux terms in the energy budget (Eq. S1). The magnitude of the sensible heat flux term is linearly related to *g_H_*, and thus, as *g_H_* increases, more energy is dissipated via sensible heat, which reduces the magnitude of the latent heat term and therefore the effect of *g_s_*. However, the boundary layer conductance also acts in series with *g_s_* to determine the magnitude of the latent heat term. For the effects of variation in *g_s_* to be manifested, there must be sufficient ability for water vapor to continue to flow across the boundary layer; otherwise, the overall moisture conductance *g_M_* is small regardless of the value of *g_s_*. When boundary layer conductance is much larger than *g_s_*, the latent heat term becomes linearly related to stomatal conductance, but in this case, VPD also decreases because the leaf temperature tends toward the air temperature. As such, results suggest that increasing boundary layer conductance via an increase in wind speed as shown in Fig. 4 tends to decrease *S*.

Stomatal conductance (*g_s_*): A considerable complication in estimating and analyzing the response of sensitivity to changing environmental conditions is that stomatal conductance itself responds to changing environmental conditions, which in turn affects sensitivity. As was illustrated graphically in Fig. 1, sensitivity (or the gradient in the *T_l_* − *g_s_* curve) decreases as stomatal conductance increases. Stomatal conductance generally increases with increasing light, decreasing VPD, and decreasing boundary layer conductance, which decreases transpiration rate [37]. The magnitude of these responses may be species or genotype-specific. This is in contrast to sensitivity at constant stomatal conductance, which increases with increasing light, increasing VPD, and decreasing boundary layer conductance. This means that as VPD increases (causing reduction in *g_s_*), sensitivity due to environmental conditions and sensitivity due to *g_s_* increase in response to environmental conditions both lead to an increase in sensitivity. However, these act in opposing directions when light or boundary layer conductance changes, and may offset to some degree. When estimating sensitivity in the field, the possibility that stomatal conductance itself might change significantly in response to environmental conditions should be considered.

The simple equations for predicting sensitivity parameters described in the “Simple equations for estimating sensitivity, *S*” section were able to give reasonable estimations of sensitivity given input environmental conditions. The resulting equations with appropriate parameters are given in Supplementary 3. The fits were able to closely reproduce the energy balance solution, as evidenced by the high coefficient of determination (*R*^2^ > 0.97), indicating that they could be used for rough estimation of *S* under different ambient conditions as represented in Fig. S1. However, it is important to note that the mathematical models were developed assuming that the sunlit and shaded leaves each had a fixed value for *R_SW_* (respectively 600 and 50 W m^−2^), yet in reality, these fluxes can vary. This assumption was necessary for simplification purposes. Despite this, the *S* values from typical *R_SW_* values in the field under clear skies around mid-day should not vary by a large margin.

#### Scaling sensitivity from single leaf to canopy

**Fig. 5.**
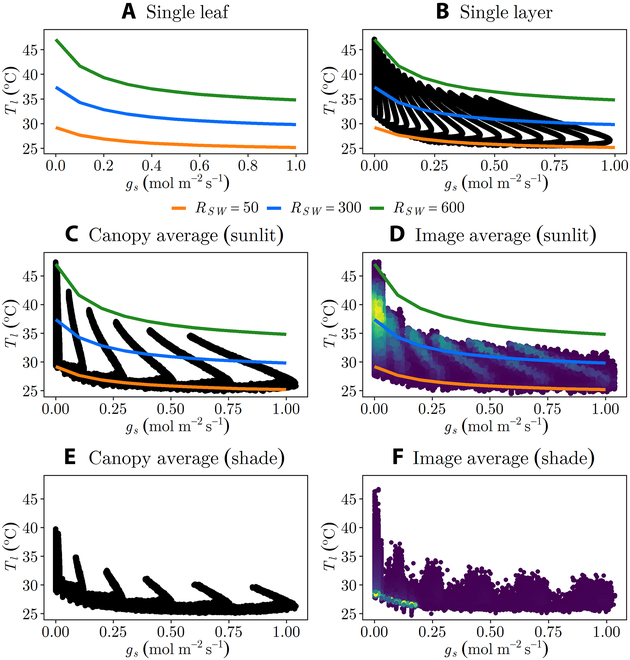
Relationship between *T_l_* and *g_s_* for different levels of aggregation at constant ambient conditions of 30^∘^C  (*T*_air_), 0.5  (*Rh*), and 1  m  s^−1^  (*U*). (A) Trend of an isolated single leaf with absorbed radiation flux *R_SW_* of 50, 300, and 600 W  m^−2^. The line plots of the single leaf were replotted in (B) to (D) to show how the single leaf *T_l_* − *g_s_* trend relates when scaling at a canopy level. Each spike shown in (B) (single layer), (C) (canopy average with sunlit leaves), and (D) (canopy average with shaded leaves) is a single *E_m_* value, which was set in the stomatal conductance model to vary the *g_s_* values. (D and F) Aerial thermal image (image average) of the homogeneous canopy for sunlit and shaded thermal pixels, respectively. The brighter yellow colors in (D) and (F) shows the highest density of thermal pixel data points.

Figure 5 illustrates the *T_l_* − *g_s_* relationship and how it varies at different levels of aggregation from an isolated single leaf to the canopy level (which includes single layer, canopy average, and image average) considering constant ambient conditions of 30^∘^*C*  (*T*_air_), 0.5  (*Rh*), and 1  m  s^−1^  (*U*). The resulting sensitivity in relation to these various aggregation scales are detailed separately below.

Single-layer canopy: Canopies consist of multiple leaves at different orientations, which create highly variable absorbed radiation fluxes. In the case of a single-layer canopy, there is no leaf shading, and thus, radiation and the *T_l_* − *g_s_* relationship vary only based on leaf orientation and the ambient environment. The single-layer canopy is therefore simply a collection of single leaves with varying *R_SW_* and all other environmental variables constant. By leveraging the *T_l_* − *g_s_* trend observed for a single leaf (Fig. 5A), we can gain insights into the general sensitivity behavior of canopies. Specifically, a single isolated leaf with absorbed shortwave radiation flux of *R_SW_* = 600  W  m^−2^ exhibits a similar trend as the maximum *T_l_* achieved by fully unobstructed leaves oriented toward the sun in the canopies (Fig. 5B). This line provides an approximate upper bound for the canopy since it is the case of a “full sun” leaf in the canopy. Similarly, a single leaf with absorbed radiation flux of *R_SW_* = 300  W  m^−2^ corresponds to the canopy average *T_l_* of the sunlit leaves (half of the canopy leaf area is projected toward the sun since *G* = 0.5 for a spherical leaf angle distribution). Also, the single leaf at *R_SW_* of 50  W  m^−2^ represents a leaf receiving only diffuse solar radiation (e.g., leaf is perpendicular to the sun or fully shaded), serving as an approximate lower bound in canopy *T_l_*. The trends of a single leaf at different *R_SW_* were also included in the sunlit canopy plots (Fig. 5 B to D) to further clarify how the *T_l_* − *g_s_* relationship behaves across different scales of measurement.

We can infer that the average sensitivity of the single-layer canopy will be similar to the single leaf with radiation flux equal to that of the average absorbed radiation flux of the single-layer canopy (i.e., *R_SW_* = 300  W  m^−2^). Therefore, the overall sensitivity of the canopy will be dependent on environmental variables discussed earlier for single leaves, and additionally the leaf angle distribution. Leaf angle distributions that increase the average absorbed radiation flux will tend to increase overall sensitivity. For example, if the sun is directly overhead and the leaf angle distribution is “planophile,” *G* = 0.85, and correspondingly, the canopy averaged absorbed radiation flux is 85% of the maximum absorbed flux [33].

**Fig. 6.**
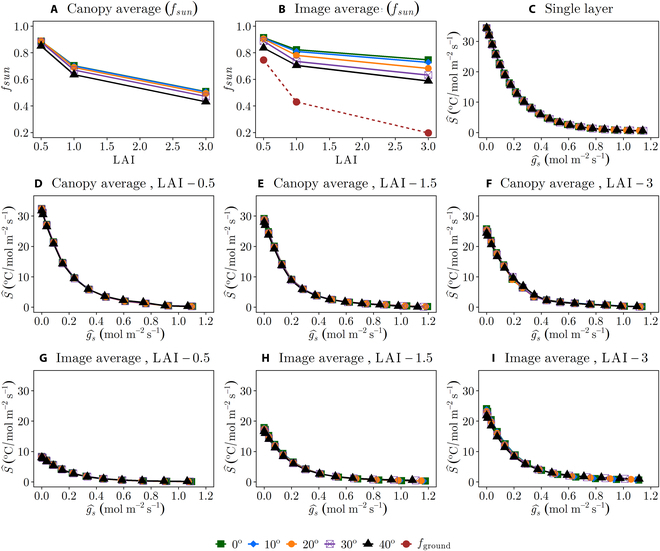
The fraction of sunlit leaves (*f*_sun_) with varying LAI is shown for (A) canopy average and (B) image average, which also includes how much of the ground is viewed in a thermal image (*f*_ground_) for different LAI (*f*_ground_ does not change with solar zenith angle). Variation in average *S* with varying *g_s_*, LAI, solar zenith, and aggregation scale is shown in (C) to (I): (C) single-layer canopy, (D) canopy average with LAI = 0.5, (E) canopy average with LAI = 1.5, (F) canopy average with LAI = 3, (G) image average with LAI = 0.5, (H) image average with LAI = 1.5, and (I) image average with LAI = 3. The single layer generally has the highest *S* because there are no shaded leaves. For the canopy averages, *S* decreases with an increase in LAI due to the increase in the fraction of shaded leaves. For the image averages, *S* increases with an increase in LAI due to the decrease in ground exposure. The hat operator denotes a spatial average.

Multilayer canopy average: A canopy with multiple layers exhibits variation in leaf absorbed radiation flux and the *T_l_* − *g_s_* relationship due to both leaf orientation and shading by other leaves. Extracting only the sunlit portion of the canopy (*η* > 0.5; Eq. 6) results in behavior that is essentially the same as the single-layer canopy (Fig. 5B versus Fig. 5C). The sunlit portion of the canopy can be viewed conceptually as a single-layer canopy. The shaded portion of the canopy tends to have lower average absorbed radiation fluxes and leaf temperatures than the sunlit portion (Fig. 5E). It will therefore tend to have lower sensitivity, and as a result, increasing the fraction of shaded leaf area will tend to decrease *S* for the overall canopy. Increasing LAI increases the fraction of shaded leaf area and thus decreases the overall *S* of the canopy (Fig. 6A). The sensitivity of the low LAI multilayer canopy is similar to the single-layer canopy (Fig. 6C), since when LAI is much less than 1.0 there is effectively only a single layer in the “multilayer canopy.” When LAI becomes large, the sensitivity of the multilayer canopy average temperature decreases substantially because of the large increase in shaded leaf area (Fig. 6 D, E and F).

Thermal image average (of multilayer canopy): The temperature viewed from an above-canopy thermal image consists of some mixture of sunlit leaves, shaded leaves, and the ground, and its sensitivity will thus be some weighted average of these components. In the scenario considered in Fig. 5D and F, the camera viewing direction is aligned with the sun direction, and therefore, the majority of leaves in view are sunlit, which is because the leaves occluded from the view of the camera will also be shaded by the sun since the two are aligned. There are still some shaded leaves in view of the camera because it is not a pinhole camera and has a field of view that includes slightly off-nadir angles. Increasing LAI increases the fraction of shaded leaf area as well as decreases the fraction of the ground surface that is in view, both of which decrease *S* in the image (Fig. 6). When LAI is low, there is a large fraction of ground in view, and thus, sensitivity is considerably reduced relative to the single-layer and multilayer canopies (Fig. 6). The ground has an *S* of zero, so any ground included in the image will decrease overall sensitivity.

### Case study: Detecting differences in stomatal traits among sorghum genotypes in a breeding trial

#### Effect of ambient conditions

**Fig. 7.**
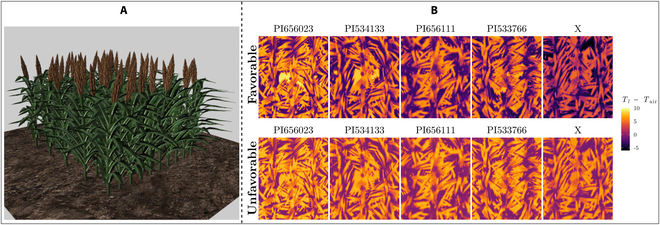
(A) Visualization of 3D model sorghum canopy. (B) Example of simulated aerial thermal images captured by the thermal camera for five sorghum genotypes under conditions of high *S* (favorable) and low *S* (unfavorable). Images show the difference between surface temperature and ambient air temperature (*T*_air_ = 40.3^∘^C for favorable, and *T*_air_ = 14.1^∘^C for unfavorable) in order to increase contrast.

Figure 7 shows an example visualization of the 3D sorghum canopy model, along with sample simulated thermal images illustrating the *T_l_* variation in favorable and unfavorable conditions. Under favorable conditions with a very large sample size, all three simulated thermal instruments (thermocouple, IR thermometer, and thermal camera) produced average *T_l_* estimates that were significantly different across genotypes (see Fig. S3). This was attributed to the large sample size and high *S* associated with favorable ambient conditions, and thus, small differences in *g_s_* could be captured by the *T_l_* measurement such that one could statistically distinguish between genotypes.

**Fig. 8.**
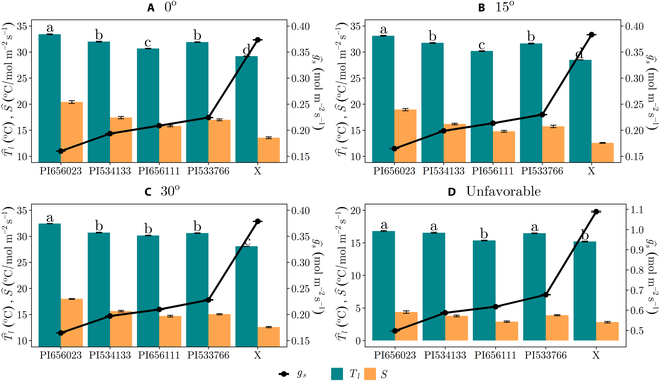
Ability of the thermal camera to detect a difference in average stomatal conductance among sorghum genotypes for varying camera viewing angles and environmental conditions. (A) to (C) compare different viewing angles at environmental conditions with moderate sensitivity: (A) 0^∘^, (B) 15^∘^, and (C) 30^∘^ between the thermal camera viewing direct and sun direction. (D) Results for a viewing direction of 0^∘^, but unfavorable conditions. Error bars indicate standard deviation. Groups sharing the same letter are not statistically different from each other at a significance level of *α*= 0.05, based on Dunn’s post hoc analysis following Kruskal–Wallis test. *S* had the same significance designations as *T_l_*, and *g_s_* is significantly different across genotypes for all cases. The hat operator denotes a spatial average.

For the case of the simulated thermal camera with viewing direction aligned with the sun direction (Fig. 8A versus Fig. 8D), changing ambient conditions from moderately favorable to unfavorable had a considerable impact on the ability to distinguish between genotypes. Under unfavorable conditions, it was not possible, for example, to distinguish between the genotypes “PI656023” and “PI533766,” and “PI656111” and “X,” despite them having very different average *g_s_* values. The inability to distinguish between “PI656111” and “X” was likely driven mostly by the very large increase in *g_s_* due to the low VPD of the unfavorable conditions. As was discussed previously, decreasing VPD decreases sensitivity directly and also increases stomatal conductance, which further decreases sensitivity.

Estimated values of sensitivity for given environmental conditions, such as by using Fig. 4 or Eq. 5, can be used to determine whether conditions are favorable for *T_l_* measurement. To illustrate this, the ambient conditions previously employed in this case study were used to estimate *S*, specifically focusing on scenarios where the thermal camera and the sun are positioned directly above the canopy. These ambient conditions include favorable (*T*_air_ = 40.3^∘^*C*, *Rh* = 0.18, *U* = 2.77 m s^−1^), moderate (*T*_air_ = 31.1^∘^*C*, *Rh* = 0.29, *U* = 4.78 m s^−1^), and unfavorable (*T*_air_ = 14.1^∘^*C*, *Rh* = 0.75, *U* = 4.74 m s^−1^) conditions. By referring to Fig. 8, it is evident that the sorghum genotypes “PI656023” and “X” in all cases represent the upper and lower bounds of *S* values, respectively, for the genotypes considered. As such, the highest *S* values for each of the ambient conditions were 40.4 (favorable), 20.4 (moderate), and 3.78^∘^C/mol  m^−2^  s^−1^ (unfavorable). Conversely, the lowest *S* values were 27.3 (favorable), 13.6 (moderate), and 2.82^∘^C/mol  m^−2^  s^−1^ (unfavorable).

#### Effect of measurement aggregation and viewing direction

For environmental conditions resulting in moderate sensitivity, the movement of the sun had a minimal effect on the ability to distinguish between genotypes using the thermocouple and IR thermometer (see Fig. S3), but it did have an impact on the thermal camera that became more important as the angle between the camera viewing direction and the sun zenith increased. When the sun was directly overhead the sorghum canopy under moderately favorable ambient conditions, the thermal camera was not able to statistically distinguish between genotypes “PI534133” and “PI533766” (Fig. 8A). Movement of the sun’s zenith to 15^∘^ relative to the thermal camera produced similar results as the previous case when the sun was directly overhead (Fig. 8B). In addition, the thermal camera was not able to statistically differentiate between three of the sorghum genotypes (“PI534133,” “PI656111,” and “PI533766”) when the sun’s zenith was 30^∘^ relative to the thermal camera. This is likely because the simulated thermocouple and IR instruments are able to largely remove the shaded leaf area and the ground, which have low or zero sensitivity. The thermal images with the viewing direction aligned with the sun direction (0^∘^) contain very few shaded leaves but may contain some ground surface area depending on the canopy structure. Furthermore, the thermal camera measurements under unfavorable conditions indicated that almost all genotypes were not significantly different from each other (Fig. 8D), which was attributed to a low *S* generated under conditions of low *T*_air_, high *Rh*, and high *U*. Based on this analysis, it can be inferred that incorporating shaded leaves in thermal camera leaf temperature (*T_l_*) measurements due to the sun’s zenith angle exceeding 30^∘^ might have a nearly equivalent adverse effect on the statistical differentiation between genotypes when compared to thermal camera measurements taken under unfavorable ambient conditions characterized by low *S*.

#### Effect of sample size

**Fig. 9.**
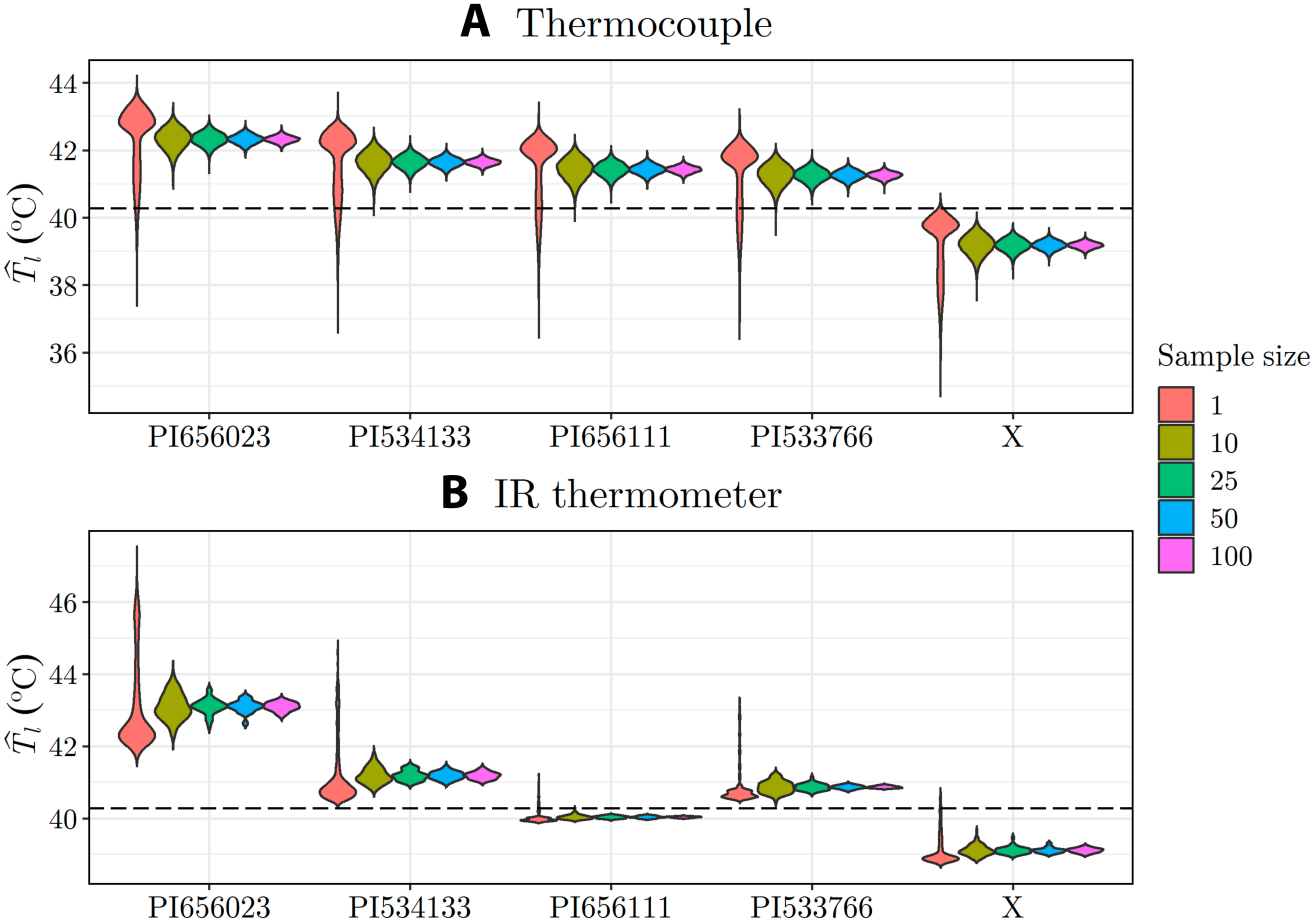
Violin plots showing the *T_l_* distribution across genotypes for (A) thermocouple and (B) IR thermometer conducted under favorable conditions (*T*_air_ = 40.3^∘^C, *Rh* = 0.18, *U* = 2.77 m s^−1^). *gs* of the genotypes decreases from left to right, and the horizontal dotted lines represent the air temperature. The hat operator on ${\hat{T}}_{l}$ denotes that average values of *T_l_* were considered in this scenario.

The impact of sample size on *T_l_* for thermocouple and IR thermometer measurements was visualized using violin plots to graphically show overlap in the probability distributions of *T_l_* at different sample sizes (Fig. 9). As expected, increasing the sample size reduced variation in the ${\hat{T}}_{l}$ distribution [38], where each “sample” in the distribution is an average of multiple measurement points. When only a single measurement was used to estimate *T_l_*, there was considerable overlap between the distributions for all genotypes with both measurement types. Using a sample size of 10, the variance in the distribution was reduced substantially such that there is little to no overlap between the distributions of genotypes with very different *g_s_*.

In general, an increase in *g_s_* is expected to correspond to a decrease in *T_l_*. This trend is clearly observed in the *T_l_* measurements obtained using the thermocouple, where the mean *T_l_* decreases with genotypes of higher *g_s_* (Fig. 9A). However, when examining the *T_l_* measurements obtained using the IR thermometer (Fig. 9B), genotype “PI656111” had a lower average *T_l_* compared to genotype “PI533766,” although it had higher average *g_s_*. This discrepancy could potentially be attributed to structural variations among the genotypes, such as differences in ground, stem, or panicle exposure during IR measurements, which is not the case for thermocouple measurements since its measurements correspond directly to the temperature of a single subleaf element [39].

The effect of sample size was more quantitatively analyzed by calculating the statistical power associated with different sample sizes [40]. The question of how many samples are needed to have an 80% or 90% probability of observing statistically significant differences (*P* < 0.05) between two groups of genotypes (i.e., statistical power) is explored in Table. Generally, the thermocouple measurements required more samples than the IR thermometer *T_l_* measurements to achieve a given statistical power, which shows that the smaller the scale of measurement, the more samples are required to represent the entire population. This is likely because the IR thermometer inherently provides some averaging that reduces the number of samples needed, and results suggest that the associated increase in statistical power outweighs the reduction in sensitivity due to the potential inclusion of nonvegetative elements like the ground.

**Table.** Table showing the sample size required to achieve a statistical power of 0.8/0.9 across genotypes for thermocouple and IR thermometer *T_l_* measurement. The Mann–Whitney *U* test could not take sample sizes less than 4, so some values were represented as “<4,” which means that there is a possibility to achieve a 0.8/0.9 statistical power with even less than four samples.

Thermocouple				
	PI534133	PI656111	PI533766	Genotype X
PI656023	23/30	16/20	14/18	<4/<4
PI534133	X	58/76	48/67	5/7
PI656111	X	X	56/77	7/8
PI533766	X	X	X	8/9
IR thermometer				
	PI534133	PI656111	PI533766	Genotype X
PI656023	8/9	<4/<4	<4/7	<4/<4
PI534133	X	5/6	40/62	<4/<4
PI656111	X	X	5/6	7/9
PI533766	X	X	X	<4/<4

Genotypes that had very different *g_s_* (e.g., “PI656023” and X) required relatively few samples to statistically differentiate them (<4 samples). Excluding genotype X, which had *g_s_* most dissimilar from other genotypes, at least 14 and as many as 58 thermocouple measurements were required to distinguish between genotypes 80% of the time, and between 18 to 77 measurements to distinguish between genotypes 90% of the time. The IR thermometer generally required fewer measurements to distinguish between genotypes. Again excluding genotype X, it required ≲4 to 40 IR measurements to distinguish between genotypes 80% of the time, and ≲4 to 62 IR measurements to distinguish between genotypes 90% of the time. Generally, the required sample size will depend on the study’s objectives, the level of accuracy desired, the magnitude of the differences among the groups, and the variability of the data.

## Discussion

### Factors influencing sensitivity at the leaf scale

The results of this study agree with [17] who reported that sensitivity of leaf temperature to stomatal conductance increases with an increase in VPD (i.e., high *T*_air_ and low *Rh*), increase in *R_n_*, and decrease in *U*. These ambient conditions leading to high sensitivity were regarded as favorable conditions and, therefore, the most preferable for the collection of *T_l_* measurements. The same recommendations align with [12] who suggested collecting *T_l_* measurements when the sky is clear, with little or no wind with low *Rh* less than 0.6, and *T*_air_ higher than 15^∘^C. For hand measurements of *T_l_* such as from an IR thermometer and thermocouple, Pietragalla and Pask [12] also suggested taking measurements in plot sections most exposed to the sun and cautioned against including operator shadows and/or shadows from neighboring plots. This is still attributed to a high *S* associated with sunlit leaves. As expected, *S* decreased with an increase in exposure of the ground in *T_l_* because the ground has *S* of 0, and thus, the ground should be avoided in *T_l_* measurements. For the IR thermometer, this could be addressed by tilting the instrument to avoid the ground [12,41]. When using a thermal camera, the leaves can be isolated from the ground using a threshold pixel segmentation approach [42] or co-registration approach, which combines thermal and other imagery such as RGB or multispectral [43,44].

Prior work has interpreted the relationship between leaf temperature and stomatal conductance in terms of the “stomatal decoupling coefficient” (Ω), introduced by [45], which characterizes the linkage between the saturation deficit at the leaf surface (*D_l_*) and that of the air outside the leaf boundary layer (*D_a_*). This coefficient helps explain the extent to which stomata regulate transpiration. When Ω = 0, there is a strong coupling between *D_l_* and *D_a_*, indicating that changes in vapor and heat fluxes from the leaf surface minimally affect the saturation deficit at the leaf surface. In this case, a fractional change in *g_s_* leads to an equivalent fractional change in transpiration. Conversely, when Ω = 1, the leaf surface conditions become completely decoupled from the air outside the leaf boundary layer, and *D_l_* approaches a local equilibrium value primarily influenced by *R_n_* and *g_s_* itself. In this scenario, a small change in *g_s_* across the entire leaf does not result in any change in transpiration rate, indicating that stomata have limited control over leaf-level transpiration. Therefore, Ω increases with increasing *g_s_*, and at high *g_s_*, stomata have minimal control over transpiration [45,46]. On the contrary to Ω, increasing *g_s_* generally increases the magnitude of the latent heat flux term and decreases *S* because the effect of an incremental increase in *g_s_* decreases at larger *g_s_* (Fig. 1). When *g_s_* is very low, transpiration is fully limited by stomata (high *S*) and a small change in *g_s_* in this regime translates to a large change in *T_l_* [47]. When *g_s_* was equal to 1 mol  m^−2^  s^−1^, *S* was very low and varying *g_s_* had almost no effect on *T_l_*.

### Interpreting and applying leaf-level sensitivity values

Graphical representations were developed to visually portray how *S* varies with ambient conditions and to provide a means for rough graphical estimation of *S* by practitioners (Fig. 4). In addition, simple mathematical models were also developed to allow more quantitative prediction of *S* given *T_l_* and ambient conditions as inputs (Supplementary 3). Once an estimation of *S* is obtained, a rough categorization of *S* values can be used to interpret its meaning and guide application. Values falling below 10^∘^C/mol  m^−2^  s^−1^ can be categorized as unfavorable conditions and should be avoided due to their low sensitivity. Meanwhile, ambient conditions corresponding to *S* values ranging from 10 to 20^∘^C/mol  m^−2^  s^−1^ may be considered moderate, with acceptability based on the particular application. *S* values surpassing 20^∘^C/mol  m^−2^  s^−1^ are considered favorable, indicating high sensitivity. It should be noted that this categorization is approximate and based on sensitivity at the leaf level, and additional factors affect sensitivity at the canopy scale as shown above. However, this categorization may be useful for practitioners in evaluating the impacts of weather conditions on the sensitivity of leaf temperature to *g_s_*.

Sensitivity values can be used to make rough estimates of the change in *g_s_* that could be detected by an instrument with a given accuracy. If the instrument accuracy is ±0.5^∘^C, which is characteristic of typical IR thermometers, it can be estimated that a ±0.05  mol  m^−2^  s^−1^ (i.e., 0.5/*S*) or larger change in *g_s_* could be reliably detected if *S* is equal to 10^∘^C/mol  m^−2^  s^−1^. It should be noted that this is only a rough estimation to give a sense of the meaning of the value of *S*.

To conduct a simple sensitivity analysis, let us consider a sunlit (*R_SW_* = 300  W  m^−2^) isolated single leaf with a *g_s_* of 0.25  mol  m^−2^  s^−1^ under moderate ambient conditions of 20^∘^C (*T*_air_), 0.4 (*Rh*), and 1  m  s^−1^ (U). Keeping other conditions constant, an increase in *T*_air_ to 30^∘^C leads to a 39% change in *S*. Also, the decrease in *R_SW_* from sunlit conditions (*R_SW_* = 300  W  m^−2^) to shaded (*R_SW_* =  50  W  m^−2^) leads to a 46% change in *S*. *Rh* and *U* yield the lowest percentage change in *S* of 16% and 20%, when changed to 0.6 and 3  m  s^−1^, respectively. Although these comparisons are not entirely relative due to the differences in the units of measurement for the ambient conditions, it can still be argued that measuring *T_l_* at high *T*_air_ for sunlit leaves takes precedence over *Rh* and *U*. This argument is supported by [19] who showed that *T_l_* variations could be over 40% when *T*_air_ changes, compared to *Rh* and *U*, which both give *T_l_* variation of about 5%.

One challenge in estimating sensitivity values is that it requires a rough idea of the range of *g_s_* for the crop species since *S* can be highly sensitive to the value of *g_s_* itself. For instance, if we consider moderate ambient conditions of 20^∘^C (*T*_air_), 0.4 (*Rh*), and 1  m  s^−1^ (*U*) for a sunlit (*R_SW_* = 300  W  m^−2^) isolated leaf, a 0.05  mol  m^−2^  s^−1^ increase in *g_s_* from 0 leads to a 1.14^∘^C change in *T_l_*. The same increase in *g_s_* (0.05  mol  m^−2^  s^−1^) from 0.5 and 1  mol  m^−2^  s^−1^ corresponds to 0.15^∘^C and 0.02^∘^C changes in *T_l_*, respectively. This clearly shows that for the same change in *g_s_*, a much higher *T_l_* change is expected at lower *g_s_* values. Consequently, detecting changes at high *g_s_* values may require extremely favorable conditions and/or a high-resolution thermal instrument to be detected. Jones [47] highlighted the importance of cautious interpretation of *T_l_* data, particularly in freezing tolerance experiments. It was noted that treatments such as salicylic acid could lead to a 0.5 to 1^∘^C *T_l_* increase attributed to salicylic acid-induced thermogenesis [48], which may wrongfully be ascribed to stomatal activity. Therefore, it is crucial to identify and account for any confounding factors that may influence changes in *T_l_*.

### Considering scale of aggregation: The trade-off between sample size and sensitivity

The scale of *T_l_* measurement can affect the sensitivity of *T_l_* to *g_s_*, as illustrated in the case study. The selection of the measurement scale by practitioners typically depends on the resources available and the size of the plot to be measured. Point temperature measurement, such as using a thermocouple, has the advantage that specific leaves can be selected for measurement that maximizes sensitivity. Leaf-level sensitivity can be relatively well understood and estimated based on the graphical or empirical approaches suggested above. Thus, by sampling leaves that are fully sunlit and on the outer canopy where the VPD is likely to be highest, sensitivity can be maximized. However, there are two primary drawbacks to this approach. One is that there is likely to be high variability between individual measurements, which thus necessitates a large sample size. Case study results suggested that even when there are very strong differences between genotype *g_s_* values, 5 to 10 measurement samples per genotype are needed to reliably distinguish between the genotypes (Table). To distinguish between genotypes with more typical differences in *g_s_*, somewhere around 20 to 50 samples per genotype are needed. The labor requirements to carry this out in a typical breeding trial may be prohibitive.

Using a measurement device that aggregates over a larger scale has the advantage that it provides inherent averaging that can reduce variability between measurements, and potentially reduce the necessary sample size. However, this comes at the cost of reducing measurement sensitivity, which is because the aggregation will unavoidably include elements with lower (or zero) sensitivity such as shaded leaves and the ground. An IR thermometer includes a moderate scale of aggregation (i.e., the beam spot size) and thus provides a moderate reduction in sample size along with a moderate reduction in sensitivity. Case study results suggested that in some cases the IR thermometer offered greater than 4× reduction in sample size relative to the thermocouple, but in other cases, the required sample size was similar (Table 1). A typical beam spot size diameter may be around 5 cm, which means that it may include multiple leaves and possibly a small portion of ground. If the user is careful in aiming the IR thermometer to include multiple fully sunlit leaves while also minimizing exposure to shaded leaves and the ground, it should be possible to reduce the required sample size relative to a point measurement due to aggregating multiple leaves while also avoiding a reduction in sensitivity due to shaded leaves or the ground. This may be difficult in practice, as it can be challenging to estimate the location and extent of the beam spot size. Instruments equipped with a visible laser sight may help with this.

The thermal camera measurement offers the largest scale of aggregation, at the cost of the largest reduction in sensitivity. A single thermal camera image may aggregate dozens or hundreds of leaves, thus allowing for an effective average across within-canopy variability. If it is possible to closely align the camera view direction with the sun direction, this minimizes the presence of shaded leaves, and thus, the main reductions in sensitivity relative to point measurements are due to leaf angle and ground surface in view. The inclusion of leaves with normals not perpendicular to the sun, which will have lower incident radiative fluxes, will tend to have lower sensitivity. Furthermore, case study results suggested that when the camera viewing direction and the sun direction differ by more than about 20^∘^, there may be a notable reduction in sensitivity due to the inclusion of shaded leaves in the image (Fig. 8).

One possible option for improving sensitivity of thermal images is to remove ground pixels from the image, either manually or using some type of thresholding approach. Deery et al. [49], for example, used the leaf temperature frequency distribution method to differentiate between ground and leaf pixels, given that dry soil temperature is usually higher than leaves. For ground surface that is fully exposed to the sun, this may be a viable option. However, shaded ground surface is likely at a similar temperature as shaded leaves, which are both likely near the air temperature. Jones and Sirault [50] mentioned that thermal measurements conducted at large scales of aggregation are associated with large pixel areas, which have a higher likelihood of containing both leaf and soil in a single pixel, making filtering difficult. With this in mind, Deery et al. [49] evaluated the impact of removing the ground from thermal imagery at a large scale, and results indicated that removing the ground did not improve the overall broad sense heritability in their wheat breeding experiment. Their experiment consisted of contrasting drought and well-watered treatments, and it is possible that the potentially large differences in *g_s_* between treatments, combined with ambient conditions with adequate sensitivity, resulted in minimal impact of the soil.

Canopy structure affects sensitivity via the leaf angle distribution, which changes the average absorbed radiation flux, and via leaf area distribution by affecting the fraction of shaded leaf area and ground surface included in the measurement. A leaf angle distribution with more leaf area projected in the direction of the sun will tend to increase the average sunlit leaf radiative flux, which will tend to increase sensitivity. Reducing total leaf area generally reduces the fraction of shaded leaf area (which has low sensitivity), but this also usually exposes more ground surface area (which has zero sensitivity). A more heterogeneous or “clumped” canopy will also tend to expose more ground surface. Reducing the scale of temperature measurement such as by using a thermocouple or IR thermometer may allow for avoiding of these canopy-level impacts by targeting portions of the canopy with high sensitivity (i.e., fully sunlit leaves on the outer canopy). As mentioned above, this is likely to come at the cost of increased sample size requirements. When LAI is low, such as early in the growing season, it may be difficult to avoid inclusion of the ground in measurements, even when using an IR thermometer. Thus, early season measurements may require either a point measurement or placing the IR thermometer very close to the plants, which in both cases is likely to cause an increase in the required sample size.

### General recommendations

Temperature measurements should be collected during periods with ambient weather conditions in which sensitivity is as high as possible, or leaf-level *S* values >10^∘^C/mol  m^−2^  s^−1^, and ideally >20^∘^C/mol  m^−2^  s^−1^. This tends to occur during periods of high solar radiation (clear skies, mid-day), high VPD (high air temperature, low humidity), and low wind speed. Leaf-level *S* values can be roughly estimated from Fig. 4 or the equations given in Supplementary 3. Depending on the application of interest, it is likely preferable to collect all measurements across time under as similar as possible weather conditions and sensitivity in order to facilitate comparison across different time points.

Regardless of the scale of measurement, for the purpose of maximizing measurement sensitivity, users should focus on targeting fully sunlit leaves. For point measurements, this simply means selecting fully sunlit leaves, and for larger-scale area-based measurements, this means maximizing the fraction of fully sunlit leaves in view of the sensor. It should also be clarified that, “fully sunlit” leaves are those in which the leaf surface is perpendicular to the sun direction, as these are the leaves that maximize incident solar flux and thus sensitivity. Leaves on the outer canopy at an oblique angle to the sun may appear fully sunlit to our eyes, but may have relatively low incident radiative fluxes due to their angle relative to the sun. The easiest way to identify fully sunlit leaves is to stand such that the sun is shining directly on the back of your head and you are looking in the same direction as the sun, then locating unobstructed leaves whose surface normals are pointing directly at you.

### Study limitations

The results presented in this study primarily stem from predictions made using a mechanistic model, and therefore, they are theoretical and should be interpreted as such. A key assumption in the case study when using the 3D biophysical model was that geometric measurements and radiative properties are homogeneous within sorghum plants of the same genotype. For geometric properties, 10 field measurements were collected for each genotype, and the average was used for inputs when developing the canopy structure. Similarly, for radiative properties, five measurements were taken for each genotype, and the average was considered as a representation of the entire genotype. This assumption was made for the sake of simplicity; otherwise, it would have required a cumbersome approach involving measurements for each plant in the field and developing each plant in a simulated environment. In addition, measuring sensitivity in the field is challenging, and determining the required sample size is complicated due to the numerous variables associated with field measurements. The models employed in this study are constrained by physics and represent the state-of-the-art in simulating various temperature measurements and their coupling with stomatal conductance. In the absence of other quantitative guidance on measurement strategies, general guidelines can be valuable for informing field practices, provided they are considered within the context of their limitations. While components of the 3D simulation framework have been validated individually [e.g., temperature/energy balance [51]; point temperature measurements (Fig. 3)], further validation of thermal imagery simulations in the field is needed in order to better understand its predictive limitations.

In order to derive simple equations for estimating *S* (i.e., Eq. 5), simplifying assumptions were needed to reduce the range of possible input values, such as the assumption that the maximum leaf-level absorbed direct radiation flux was 600 W  m^−2^ and that the absorbed diffuse radiation flux was 50 W  m^−2^. Thus, values extracted from Fig. 4 and the equations in Supplementary 3 should be taken as approximate but should provide reasonable estimates in conditions of clear skies around mid-day.

This study focused on the limitations and considerations for using temperature measurements as a proxy for stomatal conductance, but the limitations of using stomatal conductance itself as a trait representative of plant water status or drought tolerance were not explicitly addressed. If we were to assume for the sake of argument that leaf temperature could be a perfect proxy for stomatal conductance, there are still many other issues in interpreting stomatal conductance measurements for plant phenotyping and water management that are beyond the scope of this article and are discussed elsewhere (e.g., [12,52]). As mentioned by [53], a multi-sensor approach could be an alternative to define stress, where thermal measurements are used in combination with other sensing techniques like spectral analysis and fluorescence.

## Conclusions

The appropriate scale of temperature measurement depends on the total number of plants/plots to be sampled, and the magnitude of *T_l_* or *g_s_* difference that needs to be resolved for the application. A smaller scale of measurement has the potential to increase sensitivity if only leaves with high sensitivity are targeted, but this also increases measurement-to-measurement variability, which generally requires a large sample size to average out. Increasing the scale of measurement can provide averaging within the measurement itself and thus reduce the required sample size, but this may reduce overall measurement sensitivity due to inclusion of low-sensitivity elements like shaded leaves and the ground. If the goal, for example, is to detect very large differences in stomatal conductance between well-watered and drought treatments, leaf-scale measurements of temperature may be a feasible choice because only a few samples per plot are likely to be sufficient (Table 1).

Larger-scale measurement devices have the potential to increase throughput by decreasing the sample size at the expense of possibly decreasing measurement sensitivity. Measurements should be collected with the instrument viewing angle within 20^∘^ of the sun direction in order to minimize visible shaded leaf area. If the instrument has a fixed viewing direction, this typically means that measurements should be collected within a couple of hours of solar noon, while biasing measurements asymmetrically toward the afternoon may help to increase sensitivity due to ambient weather conditions.

## Data Availability

Available upon request from the authors.

## Supplementary Materials

Supplementary 1 Supplementary 1 to 3 Figs. S1 to S3 Tables S1 and S2 References (*54*–*59*)
