# Supplementary material for: Practical Considerations and Limitations of Using Leaf and Canopy Temperature Measurements as a Stomatal Conductance Proxy: Sensitivity across Environmental Conditions, Scale, and Sample Size
Source: Plant Phenomics. 2024 Apr 15;6:0169. doi: 10.34133/plantphenomics.0169 (PMC11018642; doi:10.34133/plantphenomics.0169)
Supplement: Supplementary 1 — to 3 Figs. S1 to S3 Tables S1 and S2 References (54–59) [file plantphenomics.0169.f1.pdf]

Supplementary Materials

Supplementary 1 Leaf energy balance model

The surface energy balance is a budget of energy fluxes crossing a control surface. Considering fluxes of radiation, sensible heat, and latent heat from both sides of a leaf leads to the leaf energy balance equation [23], which can be solved iteratively for the leaf surface temperature T_l

$$\underbrace{R_{SW} + R_{LW} - 2\sigma T_l^4}_{\text{Net radiation}} = \underbrace{c_p g_H (T_l - T_{air})}_{\text{Sensible heat}} + \underbrace{\lambda g_M \left(\frac{e_l - e_a Rh}{P_{atm}} \right)}_{\text{Latent heat}}, \tag{S1}$$

where: R_{SW} $(W\,m^{-2})$ is the absorbed shortwave/solar radiation flux, R_{LW} $(W\,m^{-2})$ is the absorbed longwave/terrestrial radiation flux, σ is the Stefan–Boltzmann constant $(5.67 \times 10^{-8}\ W\,m^{-2}\,K^{-4})$. c_p is the heat capacity of air $(25.95\,J\,mol^{-1}\,K^{-1})$, g_H $(mol\,m^{-2}\,s^{-1})$ is the boundary layer conductance to heat from the surface to the outside of the boundary layer, T_{air} is the absolute temperature of the air immediately outside of the leaf boundary layer, and Rh is the air relative humidity immediately outside of the leaf boundary layer. λ is the latent heat of vaporization of air $(44,000\,J\,mol^{-1})$, g_M $(mol\,m^{-2}\,s^{-1})$ is the conductance to water vapor between the leaf sub-stomatal cavity and outside of the leaf boundary layer. $e_l\,(Pa)$ and $e_a\,(Pa)$ are the air saturation vapor pressures evaluated at the leaf and air temperature, respectively, which were calculated using the Tetens equation [23]. $P_{atm}\,(Pa)$ is the atmospheric pressure $(101,000\,Pa)$. The term $\frac{e_l - e_a Rh}{P_{atm}}$ represents the VPD, and the product of the VPD and g_M is the transpiration rate.

The absorbed all-wave radiation flux R consists of absorbed incoming shortwave (R_{SW}) and long wave radiation (R_{LW}) fluxes. Incoming long wave radiation was estimated as follows

$$R_{LW} = \varepsilon_s \sigma T_{air}^4 + \varepsilon_g \sigma T_g^4, \tag{S2}$$

where ε_g is the emissivity of the ground (assumed equal to 1), T_g is the ground temperature, calculated by the ground energy balance equation (described below). ε_s is the effective emissivity of the sky and was calculated according to [54] as

$$\varepsilon_s = 1 - (1 + p)\exp\left(-(1.2 + 3p)^{0.5}\right), \tag{S3}$$

where p (cm of precipitable water) is the atmospheric water vapor path length estimated according to [55]. The above formulation of R_{LW} stated in Eq. S2 effectively assumes that the leaf is isolated in space, and thus receives half of its long wave radiation from the ground, and the other half from the sky. For more realistic plant geometries (i.e., that of the case study), the R_{LW} was calculated using a 3D radiative transfer model (see below).

The leaf boundary layer conductance to heat was calculated using the Polhausen Equation (Eq. S4)

$$g_H = 2 \times 0.135\sqrt{\frac{U}{L}}, \tag{S4}$$

where $U\,(m\,s^{-1})$ is the wind speed outside the leaf boundary layer, assumed to be perpendicular to the leaf surface, $L\,(m)$ is the characteristic dimension, which in this case corresponds to the width across the widest part of a patch or leaf. The factor of 2 accounts for convective heat transfer from both surfaces of the leaf, which is symmetric for both sides under the assumption of fully forced convection.

The semi-mechanistic model of [56] was used to estimate the stomatal conductance, which was calculated as

$$g_s = \frac{E_m(Q + i_o)}{k + bQ + (Q + i_o)VPD}, \tag{S5}$$

where Q is the photon flux density ($\mu mol/m^2 s$), E_m is the maximum transpiration rate at high VPD, i_o relates to nocturnal transpiration, k and b are empirical parameters relating to leaf-specific hydraulic conductance and turgor to conductance scalar.

The boundary layer conductance to moisture g_M consists of two serial pathways for water vapor transport from the leaf: one across the stomatal pore (associated with the stomatal conductance g_s), and another across the leaf boundary layer (associated with the boundary layer conductance to water vapor $1.08g_H$). The boundary layer conductance to moisture was estimated to be 1.08 times that of the boundary layer conductance to heat, according to the approximate ratio of diffusivity of water vapor to heat in the air. By combining the two conductances in series, and assuming that stomata are primarily located on the lower leaf surface, the overall conductance to water vapor is

$$g_M = \frac{(1.08g_H/2)g_s}{(1.08g_H/2) + g_s}. \tag{S6}$$

Equation S1 was iteratively solved for the leaf temperature T_l using the secant method [57]. For each set of ambient conditions, g_s was varied from 0 and $1\, mol\, m^{-2}\, s^{-1}$ and the resulting surface temperature was computed and fitted to Eq. 2 to derive the value of c, where T_{dry} and T_{wet} are the leaf surface temperatures when g_s is 0 and $1\, mol\, m^{-2}\, s^{-1}$, respectively.

The above methodology was used for the calculation of leaf surface temperature and was adapted to estimate ground temperature (T_g) and the temperatures of non-leaf plant components (e.g., panicle, stem) considered in the subsequent case study. Notably, the ground is treated as a one-sided surface, meaning it emits and absorbs radiation on one surface, therefore the net radiation flux (left-hand side of Equation S1) becomes $R - \sigma T_g^4$, where T_g is the ground temperature. In addition, the ground boundary layer conductance was calculated as [58]

$$g_H = 0.166 + 0.5U. \tag{S7}$$

Supplementary 2 Description of thermal camera operation in Helios using ray tracing approach

Ray tracing is a powerful computational technique that is commonly used in fields such as computer graphics and optics to simulate the behavior of light or other forms of radiation as they interact with surfaces and objects. By tracing the path of individual rays of light or radiation as they interact with different materials and surfaces, ray tracing can accurately predict complex effects such as reflections, refractions, and shadows [31]. In Helios, the ray tracing approach was used to track the path of individual rays of thermal radiation as they are emitted from a source and interact with different surfaces eventually reaching the thermal camera. A pixel was then assigned to each ray and the temperature of each pixel was calculated using Eqn S2, which relates the intensity of thermal radiation emitted by an object to its temperature. Then the pixels were combined to form a thermal image representing the temperature distribution of the surface.

Supplementary 3 Simple equations for estimating bulk sensitivity parameters in Eq. 3

The $S-parameters$ for predicting S based on simple equations are given below based on separate fits to the energy balance equation for sunlit and shaded leaves

$$\begin{aligned} c(Sunlit) = -(0.001T_{air} + 0.002U + 0.006)Rh - (0.001U + 0.002)T_{air} + \\ 0.06U + 0.274, \end{aligned} \tag{S8}$$

$$c\,(Shade) = -(0.001T_{air} + 0.015)Rh - (0.001U + 0.002)T_{air} + 0.061U + 0.303, \tag{S9}$$

$$T_{\Delta}\,(Sunlit) = [-(0.009U + 0.205)T_{air} + 0.002U - 0.776]Rh - (0.005U - 0.315)T_{air} - 0.12U + 0.781, \tag{S10}$$

$$T_{\Delta}\,(Shade) = [-(0.006U + 0.236)T_{air} + 0.032U - 1.008]Rh + (0.001U + 0.26)T_{air} + 0.052U + 0.452, \tag{S11}$$

$$T_{wet}\,(Sunlit) = [-(0.002U - 0.299)T_{air} - 0.088U + 1.479]Rh + (0.008U + 0.663)T_{air} - 0.681U + 4.787, \tag{S12}$$

$$T_{wet}\,(Shade) = [-(0.005U - 0.331)T_{air} - 0.142U + 1.774]Rh + (0.001U + 0.773)T_{air} + 0.245U - 2.911. \tag{S13}$$

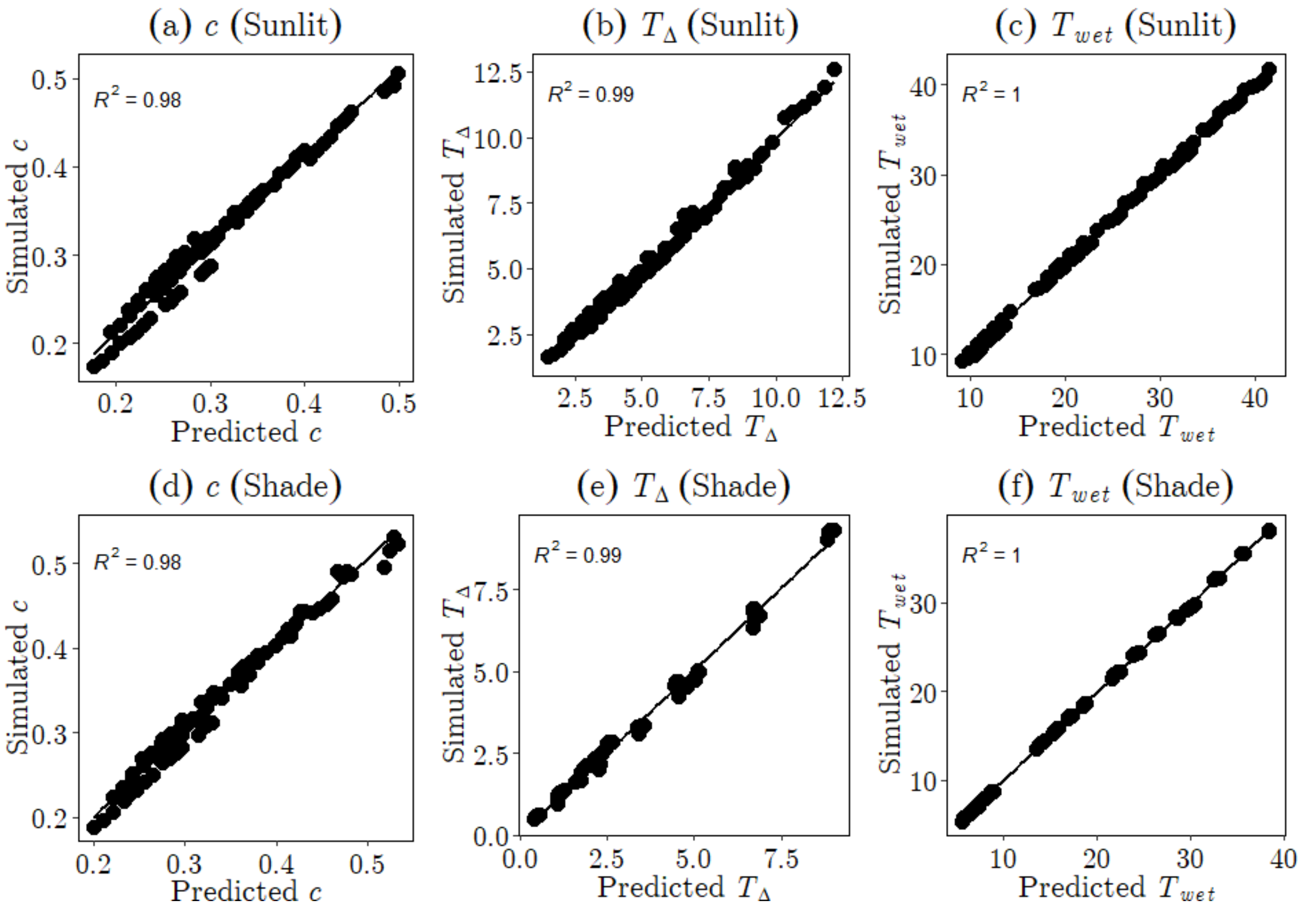

Figure S1: A linear fit showing simulated and predicted values of S-parameters with corresponding R^2 values. Overall, the R^2 values were greater than 0.97 for all scenarios, which shows that the equations have a good prediction.

To develop mathematical models for S-parameters, the Helios framework was used to generate T_l values for variations in $g_s\,(0 - 1\,mol\,m^{-2}\,s^{-1})$, $T_{air}\,(10 - 40\,^{\circ}C)$, $Rh\,(0.2 - 0.8)$, and $U\,(1 - 5\,m\,s^{-1})$ for both sunlit and shaded leaves. These T_l and g_s values were plotted to generate Fig. 1, and the curve was fitted to Eq.2 to obtain S-parameter values (c, T_{wet}, and T_{Δ}) for each combination of ambient conditions. Considering one S-parameter at a time, data

sets with the same U were grouped together, and for each group, plots of S-parameter ($y-axis$) against $Rh(x-axis)$ were made and linearly fitted to a form of "$a_1 Rh + a_2$", where a_1 and a_2 are coefficients that represent a list of slope and y-intercept values at different T_{air} for each group, respectively. This procedure of linear fitting and generating the corresponding slope and y-intercept was repeated for each coefficient until all the coefficient values were generated, yielding Eq. 5 as the output with the ambient conditions as inputs. These steps are summarized in Fig. S2.

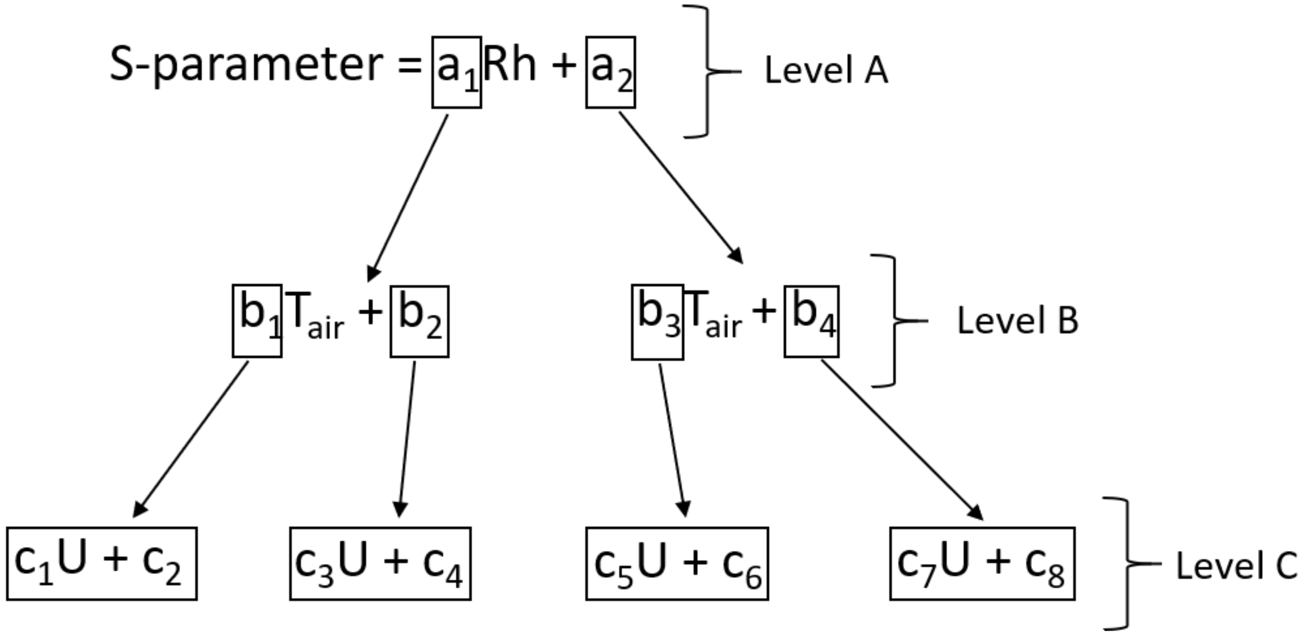

Figure S2: The steps for generating equations of S-parameters. The data set was linearly fitted to generate the corresponding slope and y-intercept. The procedure was repeated up to level C where all the coefficient values were generated.

Table S1: Summary of the sorghum genotypes used in the case study [32, 59]

**Genotype**	**Origin**	**Type**	**Working group**	**Panicle description**
PI656023 (Segaolane)	Botswana	Selected landrace	Kafir	Erect, elongated, mostly semi-compact and cylindrical
PI534133 (SC35)	Ethiopia	Converted landrace	Durra	Stiff, dense and compact, ovate to oblong
PI656111 (SC971)	Puerto Rico	Converted landrace	Durra-Kafir	Long, semi-compact with branches with a stout base
PI533766 (SC265)	Burkina Faso	Converted landrace	Guinea (Conspicuum)	Hairy, rarely glabrous, long and pendulous

Table S2: Summary of field measurements used to develop the sorghum 3D structures in Helios. A stem radius of 0.011 m was used for all genotypes.

**Genotype**	**Leaf length (m)**	**Leaf width (m)**	**Stem height (m)**	**Panicle height (m)**	**Panicle diameter (m)**	**Number of leaves**	E_m ($mmol\, m^{-2}\, s^{-1}$)
PI656023	0.5483	0.0596	0.753	0.205	0.0475	16	6.4
PI534133	0.5151	0.0564	1.120	0.175	0.0625	18	7.4
PI656111	0.5566	0.0589	1.315	0.162	0.103	21	7.9
PI533766	0.5489	0.0623	0.924	0.165	0.086	20	8.4
Genotype X	0.5422	0.0593	1.028	0.177	0.0748	19	12.5

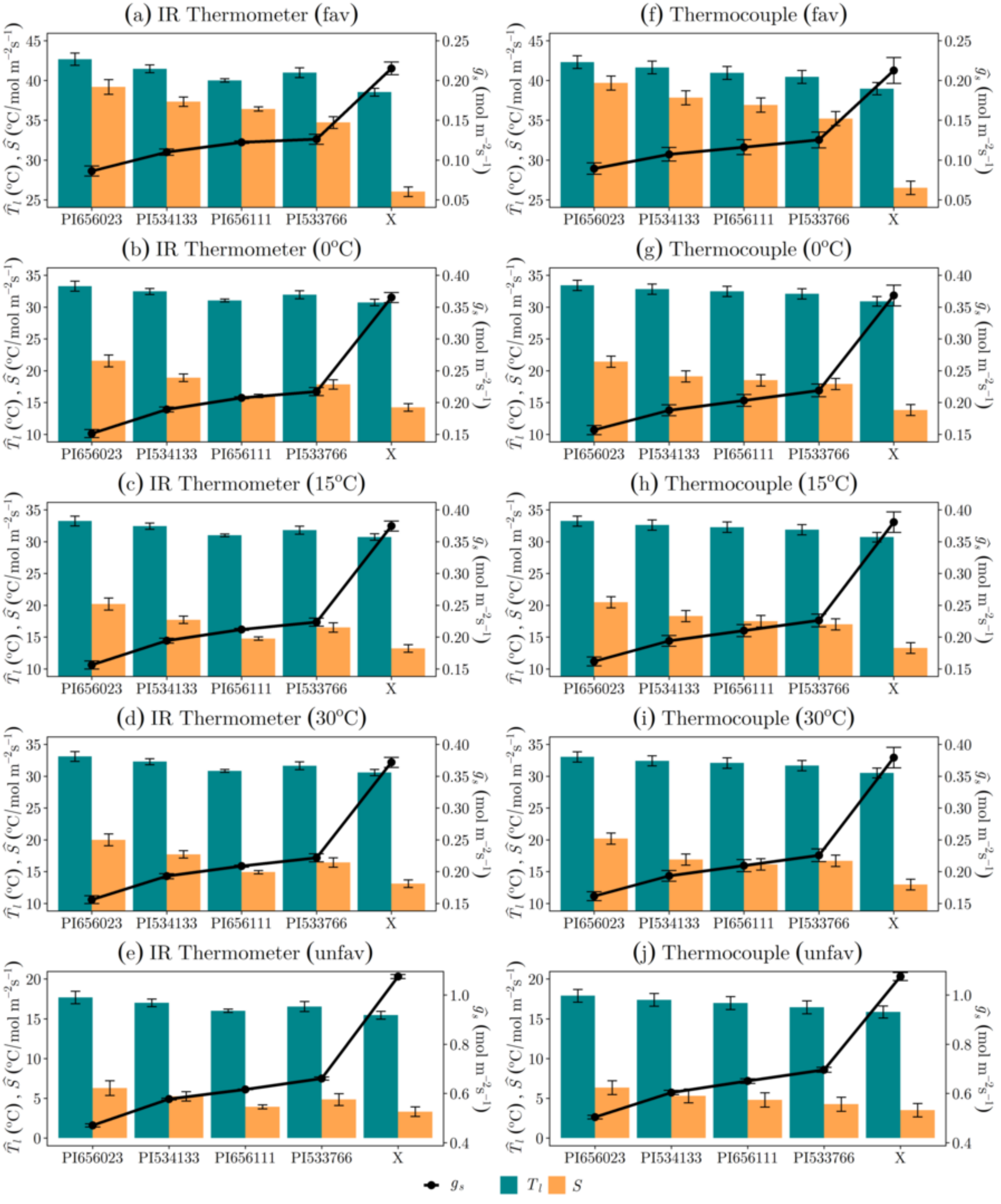

Figure S3: Comparing leaf temperature and sensitivity responses to stomatal conductance across five genotypes under various viewing angles and environmental conditions for (a-e) Infrared thermometer and (f-j) Thermocouple.
